# Exploring Syndecan-4 and MLP and Their Interaction in Primary Cardiomyocytes and H9c2 Cells

**DOI:** 10.3390/cells13110947

**Published:** 2024-05-30

**Authors:** Thea Parsberg Støle, Marianne Lunde, Katja Gehmlich, Geir Christensen, William E. Louch, Cathrine Rein Carlson

**Affiliations:** 1 Institute for Experimental Medical Research, Oslo University Hospital and University of Oslo, 0450 Oslo, Norway; marlu@medisin.uio.no (M.L.); geir.christensen@medisin.uio.no (G.C.); w.e.louch@medisin.uio.no (W.E.L.); c.r.carlson@medisin.uio.no (C.R.C.); 2 K.G. Jebsen Center for Cardiac Research, University of Oslo, 0313 Oslo, Norway; 3 Institute for Cardiovascular Sciences, University of Birmingham, Edgbaston, Birmingham B15 2TT, UK; k.gehmlich@bham.ac.uk; 4 Division of Cardiovascular Medicine, Radcliffe Department of Medicine and British Heart Foundation Centre of Research Excellence Oxford, University of Oxford, Oxford OX3 9DU, UK

**Keywords:** syndecan-4, MLP, CSRP3, cardiomyopathy, oligomerization

## Abstract

The transmembrane proteoglycan syndecan-4 is known to be involved in the hypertrophic response to pressure overload. Although multiple downstream signaling pathways have been found to be involved in this response in a syndecan-4-dependent manner, there are likely more signaling components involved. As part of a larger syndecan-4 interactome screening, we have previously identified MLP as a binding partner to the cytoplasmic tail of syndecan-4. Interestingly, many human MLP mutations have been found in patients with hypertrophic (HCM) and dilated cardiomyopathy (DCM). To gain deeper insight into the role of the syndecan-4–MLP interaction and its potential involvement in MLP-associated cardiomyopathy, we have here investigated the syndecan-4–MLP interaction in primary adult rat cardiomyocytes and the H9c2 cell line. The binding of syndecan-4 and MLP was analyzed in total lysates and subcellular fractions of primary adult rat cardiomyocytes, and baseline and differentiated H9c2 cells by immunoprecipitation. MLP and syndecan-4 localization were determined by confocal microscopy, and MLP oligomerization was determined by immunoblotting under native conditions. Syndecan-4–MLP binding, as well as MLP self-association, were also analyzed by ELISA and peptide arrays. Our results showed that MLP-WT and syndecan-4 co-localized in many subcellular compartments; however, their binding was only detected in nuclear-enriched fractions of isolated adult cardiomyocytes. In vitro, syndecan-4 bound to MLP at three sites, and this binding was reduced in some HCM-associated MLP mutations. While MLP and syndecan-4 also co-localized in many subcellular fractions of H9c2 cells, these proteins did not bind at baseline or after differentiation into cardiomyocyte-resembling cells. Independently of syndecan-4, mutated MLP proteins had an altered subcellular localization in H9c2 cells, compared to MLP-WT. The DCM- and HCM-associated MLP mutations, W4R, L44P, C58G, R64C, Y66C, K69R, G72R, and Q91L, affected the oligomerization of MLP with an increase in monomeric at the expense of trimeric and tetrameric recombinant MLP protein. Lastly, two crucial sites for MLP self-association were identified, which were reduced in most MLP mutations. Our data indicate that the syndecan-4–MLP interaction was present in nuclear-enriched fractions of isolated adult cardiomyocytes and that this interaction was disrupted by some HCM-associated MLP mutations. MLP mutations were also linked to changes in MLP oligomerization and self-association, which may be essential for its interaction with syndecan-4 and a critical molecular mechanism of MLP-associated cardiomyopathy.

## 1. Introduction

The heart’s ability to withstand changes in biomechanical stress is highly dependent on signal sensing and transduction. It is, therefore, likely that transmembrane proteins are involved in this adaptive and eventually remodeling response. Syndecan-4 is a ubiquitously expressed transmembrane proteoglycan that is able to sense extracellular stress and transmit these signals intracellularly to facilitate molecular signaling [1]. Syndecan-4 localizes to the costameres/Z-discs in cardiomyocytes and is suggested to be a biomechanical stress sensor in the heart upon pressure overload [2,3]. Although the loss of syndecan-4 yields no overt phenotype in the male heart at baseline, its mRNA expression and protein levels increase upon biomechanical stress or cardiac injury such as pressure overload and myocardial infarction (MI) [2,4,5]. A role for syndecan-4 has been found in angiogenesis, hypertrophy development, inflammation, and response to MI (for reviews see [6,7,8]).

Much like the other members of the syndecan family, syndecan-4 is composed of an extracellular domain decorated with various glycosaminoglycan (GAG) chains, a transmembrane domain, and a short cytoplasmic tail [9]. In addition, the extracellular domain of syndecan-4 can be shed, yielding a soluble proteoglycan, which has been found to be increased in various physiological and pathological situations [10,11,12,13]. The intracellular tail of syndecan-4 holds no intrinsic enzymatic activity and is, therefore, highly reliant on its protein interactions to exert physiological effects.

Using an affinity purification-mass spectrometry approach, we have previously found that the intracellular tail of syndecan-4 interacts with Muscle LIM Protein (MLP), encoded by the *Cysteine and Glycine-Rich Protein 3* (CSRP3) gene [14]. MLP is a 194 amino acid-long protein belonging to the LIM-only family of proteins [15,16]. Compared to its other family members, cysteine-rich protein (CRP) 1 and 2, MLP is exclusively expressed in cardiac and skeletal muscle and first appears at the onset of myogenic differentiation in the embryo [17]. Whereas skeletal MLP expression significantly declines two weeks after birth, cardiac MLP levels do not change during post-natal development [18], indicating that the protein is likely indispensable for cardiac structure and function at these stages. Indeed, the MLP knockout (KO) mouse has a low survival rate at post-natal days 5–10 due to severe dilated cardiomyopathy [18].

As with other Z-disc proteins, MLP has been proposed to be a stress transducer in cardiomyocytes [18,19,20]. This function is likely, at least in part, maintained through MLP’s interactions with multiple protein partners. The N-terminus binds to the Z-disc protein titin-cap/telethonin (TCAP) [21], and the MLP–TCAP binding forms part of a larger complex together with titin, calcarcins, and minK, constituting part of the stretch-sensing machinery of cardiomyocytes [22,23]. Bordering MLP’s N-terminus is LIM1, the first of two LIM domains, which are known for their extensive protein-binding ability [24,25]. Two zinc fingers within these LIM domains coordinate zinc molecules, providing stable protein folding [26]. Next, a nuclear localization signal (NLS), a motif traditionally recognized by proteins belonging to the importin group [27], is likely partially responsible for the nuclear translocation of MLP. Following the NLS motif, a 12 amino acid cofilin-2 (CFL2) binding domain is found, of which the binding significantly impacts F-actin depolymerization [28]. Lastly, the second LIM domain is found (LIM2) towards the C-terminus.

Syndecan-4 and MLP interact with one another in cardiac muscle, and this interaction increases following pressure overload induced by aortic banding [14], indicating the potential importance of the interaction in disease. However, it remains unclear whether this interaction occurs within a particular subcellular fraction and the reciprocal binding sites of the two proteins. Interestingly, MLP is able to oligomerize, and its oligomeric state has been suggested to determine its subcellular localization [29]. Multiple human mutations in MLP have been found in patients with familial hypertrophic cardiomyopathy (HCM) and dilated cardiomyopathy (DCM) [19,20,21,30,31,32,33,34]. Although the molecular mechanisms of MLP-associated cardiomyopathy are not known, they likely, at least in part, involve alterations in protein interactions and the oligomerization state, and perhaps a change in subcellular localization.

To further characterize the syndecan-4–MLP interaction sites, MLP oligomerization, and the effect of human cardiomyopathy-associated MLP mutations, we have presently explored MLP, syndecan-4, and their interaction in adult primary rat cardiomyocytes and the H9c2 rat cardiomyoblast cell line. Our results show that syndecan-4 and MLP bind in isolated adult primary rat cardiomyocytes and that this occurs specifically within nuclear-enriched fractions. Despite a thorough investigation of the syndecan-4–MLP interaction in the H9c2 cell line, the binding was not present in this cell system. Additionally, through in vitro work, we find that MLP oligomerization is altered when MLP is mutated, resulting in an increase in monomeric recombinant MLP at the expense of higher-order oligomeric MLP. Our results suggest that the sites of MLP self-association are mainly located at the end of the LIM1 through the NLS and in the middle of the LIM2 domain, and self-association is altered in the presence of HCM- and DCM-associated MLP mutations.

## 2. Methods

### 2.1. Plasmids, Peptides, and Recombinant Proteins

All human MLP gene constructs (GenScript Biotech Corp, Piscataway, NJ, USA) were in a pcDNA3.1 vector, and human syndecan-4 in a pCEP4 vector. Empty vectors were used as controls. Where indicated, tagged constructs were used, where the FLAG-tag in human MLP was placed at the C-terminus, and the HA-tag in human syndecan-4 was placed between amino acids 27 and 28 at the N-terminus.

All human peptides and recombinant proteins were synthesized with >80% purity by GenScript. All MLP recombinant proteins were full length and had 6xHis tags at the N-terminus (GenScript Biotech Corp, Piscataway, NJ, USA). MLP-WT peptide sequences were synthesized as 30-mers with or without an N-terminal biotin tag. MLP mutations (in bold) were synthesized within these sequences:

MLP-W4R: MPN**R**GGGAKCGACEKTVYHAEEIQCNGRSF

MLP-L44P: CNGRSFHKTCFHCMACRKA**P**DSTTVAAHES

MLP-S46R: CNGRSFHKTCFHCMACRKALD**R**TTVAAHES

MLP-S54R,E55G: AAHE**RG**IYCKVCYGRRYGPKGIGYGQGAGC

MLP-C58G: AAHESEIY**G**KVCYGRRYGPKGIGYGQGAGC

MLP-R64C: AAHESEIYCKVCYG**C**RYGPKGIGYGQGAGC

MLP-Y66C: AAHESEIYCKVCYGRR**C**GPKGIGYGQGAGC

MLP-K69R: AAHESEIYCKVCYGRRYGP**R**GIGYGQGAGC

MLP-G72R: AAHESEIYCKVCYGRRYGPKGI**R**YGQGAGC

MLP-Q91L: QGAGCLSTDTGEHLGL**L**FQQSPKPARSVTT

Biotin-ahx-SDC4_cyt_: RMKKKDEGSYDLGKKPIYKKAPTNEFYA

Biotin-ahx-SDC4_scram_: GTKYPKMDRGKLFKYKAKPEDNESAYIK

SDC4 blocking peptide: DLGKKPIYKKAPTN

### 2.2. Peptide Arrays

Human, rat, and mouse MLP were synthesized as overlapping 20-mers with three amino acid offsets onto cellulose membranes by an automated MultiPep peptide synthesizer (INTAVIS Bioanalytical instruments AG, Tübingen, Germany). After blocking, membranes were incubated with a biotin-ahx-SDC4_cyt_ (SDC4_cyt_) peptide, a scrambled biotin-ahx-SDC4_cyt_ (SDC4_scram_), a custom-made MLP antibody (#R06991, GenScript Biotech Corp, Piscataway, NJ, USA), or a commercially available MLP antibody (sc-166930, Santa Cruz, Dallas, TX, USA) overnight at 4 °C. The remaining consequent steps were identical to those performed for immunoblotting, as described further below.

### 2.3. Enzyme-Linked Immunosorbent Assay (ELISA)

96-well ELISA microplates were coated with 10 µg of WT or mutated human 6xHis-MLP recombinant proteins or 10 µg of MLP peptides in PBS, and rotated at 4 °C overnight. Each well was rinsed with 0.05% Tween-20 in PBS (PBS-T) and blocked with 0.5% gelatin (G-1890, Sigma Merck, Darmstadt, Germany) for one hour at room temperature before incubation with the given biotinylated peptides at a final concentration of 2–5 µM for two hours at 37 °C with gentle agitation. Each well was then thoroughly washed with PBS-T five times before incubation with 100 µL of Biotin-HRP (A0185, Sigma Merck, Darmstadt, Germany) for 30 min at room temperature with gentle agitation. Each well was then washed with PBS-T 5 times before incubation with 100 µL of Ultra TMB substrate solution (34028, Thermo Fisher Scientific, Waltham, MA, USA) for 15–30 min at room temperature with gentle agitation to develop a blue color signal. The reaction was stopped with 100 µL of 2 M hydrochloric acid (HCl) per well (yellow color development). The absorbance of each well was read at 450 nm (Hidex sense multimodal microplate reader, Åbo, Finland). 

### 2.4. Biacore Surface Plasmon Resonance

A streptavidin (SA) chip (BR1000032, Cytiva, Marlborough, MA, USA) was conditioned with three 1 min injections of 1 × Biacore running buffer (BR100826, Cytiva, Marlborough, MA, USA), and biotinylated SDC4_cyt_ was immobilized at 133–350 resonance units (RUs). Recombinant 6xHis-MLP-WT was dialyzed into 1 × Biacore running buffer, and increasing concentrations were injected over the chip’s surface at a flow rate of 30 µL per minute for 180 s. For the different runs, concentration ranges spanned 2.9–14.6 nM, 1.2–100 nM, and 21.2–500 nM. The dissociation time was set to 600 s. Sensorgrams were analyzed using the Biacore X100 software (Version 2.0.1) with the assumption of 1:1 binding (Langmuir binding model).

### 2.5. Culture, Transfection, and Differentiation of H9c2 Cells

H9c2 cells (ATCC^®^ CRL-1446^TM^, Manassas, VA, USA) were cultured at 37 °C and 5% CO_2_ in a humidified incubator in DMEM media (#41965, Gibco, Thermo Fisher Scientific, Waltham, MA, USA) supplemented with 10% FBS and 1% penicillin/streptomycin (P0781, Sigma Merck, Darmstadt, Germany). For maintenance, cells were passaged at 90% confluency.

For transfection, cells were plated in 10 cm^2^ culture-grade dishes or on 12 mm Ø glass coverslips (#0117520, Paul Marienfeld GmbH & Co, Lauda-Königshofen, Germany) to a confluency of 80%. After 24 h, cells were transfected with the given constructs using the PolyJet in vitro transfection reagent (SignaGen laboratories, Frederick, MD, USA) according to the manufacturer’s protocol.

For differentiation, cells were plated in 10 cm^2^ culture-grade dishes or on 12 mm Ø glass coverslips (#0117520, Paul Marienfeld GmbH & Co, Lauda-Königshofen, Germany) to a confluency of 70%. After 24 h, the media was changed to DMEM media (#41965, Gibco, Thermo Fisher Scientific, Waltham, MA, USA) supplemented with 1% FBS and 1% Penicillin/streptomycin. Ten nM all-trans-retinoic acid (#R2625, Sigma Merck, Darmstadt, Germany) was added to the cultures daily, with media changes conducted every other day for 5 consecutive days. Where indicated, isoprenaline (ISO) was added on the sixth day for 24 h at a concentration of 25 µM (#l5627, Sigma Merck, Darmstadt, Germany).

### 2.6. Culture of HL-1 Cells

HL-1 cells [35] were cultured in flasks coated with 0.02% gelatin (G9391, Sigma Merck, Darmstadt, Germany) and 5 µg/mL fibronectin (F1141, Sigma Merck, Darmstadt, Germany) in Claycomb medium (51800C, Sigma Merck, Darmstadt, Germany) supplemented with 10% FBS, 1% penicillin/streptomycin (P0781, Sigma Merck, Darmstadt, Germany), 0.1 mM Norepinephrine (A0937, Sigma Merck, Darmstadt, Germany), and 2 mM L-Glutamine (G7513, Sigma Merck, Darmstadt, Germany) at 37 °C and 5% CO_2_ in a humidified incubator. For maintenance, cells were passaged at 90%.

### 2.7. Animal Experiments

All animal work was performed in accordance with the approval of the National Regulation of the use of animal research and the Norwegian Animal Welfare Act (FOTS ID 30114 for rats, ID IV 1-17U for neonatal rats, and ID 29268 and 23008 for mice). For left ventricular (LV) cardiomyocyte isolations, 2–4-month-old male Wistar rats were used (Janvier Labs, Le Genest-Saint-Isle, France). For WT and cardiomyocyte-specific syndecan-4 overexpressing (TG) mouse LV lysates [36], adult female mice with a C57BL/6J background were used (Jackson Laboratory, Bar harbor, ME, USA). Animals were housed in a temperature-controlled facility with 12:12 h light/dark cycles. All animals had access to food and water ad libitum and were sacrificed by cardiac excision during deep surgical anesthesia by isoflurane inhalation.

### 2.8. Adult and Neonatal Rat Left Ventricular Cardiomyocyte Isolation

The excised adult hearts were cannulated through the aorta on a constant flow Langendorff perfusion system. To clear the blood from the coronary arteries, the hearts were first flushed with approximately 5–10 mL of isolation buffer (130 mM NaCl, 5.4 mM KCl, 0.5 mM MgCl_2_, 0.4 mM NaH_2_PO_4_, 25 mM HEPES, and 5.5 mM d-glucose, pH 7.4) at a rate of 3 mL/minute. Isolation buffer supplemented with 2mg/mL collagenase type II (Worthington Biochemical Corporation, Lakewood, NJ, USA) was then perfused through the heart for 10–12 min at 37 °C. After digestion, the LV was dissected, minced, and separated into chunks in an isolation buffer containing 1 mg/mL of BSA. To further detach the cells from surrounding tissue, the chunks were transferred to a falcon tube containing 0.2 mg DNase (LS002006, Worthington Biochemical Corporation, Lakewood, NJ, USA), collagenase, and 250 µL of BSA. The cells were subsequently filtered through a 200 µm filter and left to sediment. The Ca^2+^ concentration was gradually increased to 0.2 mM. Isolated cardiomyocytes were used within 2 h of isolation. Neonatal rat cardiomyocytes were isolated as previously described [10].

### 2.9. Subcellular Fractionation

Freshly isolated rat LV cardiomyocytes and H9c2 cells were fractioned into cytoplasmic-, membrane-, nuclear-, and cytoskeletal-enriched subcellular compartments according to the manufacturer’s protocol (#2145; Merck Millipore, Burlington, MA, USA).

### 2.10. Immunoprecipitation

For the immunoprecipitations, lysates were incubated with 2 µg of syndecan-4 (KY/8.2, #550350, BD Biosciences, Franklin Lakes, NJ, USA), MLP (#R06991, GenScript Biotech Corp, Piscataway, NJ, USA) or HA-tag (#3724, Cell Signaling technology, Danvers, MA, USA) antibodies and protein A/G agarose beads (sc-2003, Santa Cruz, Dallas, TX, USA) overnight at 4 °C. The samples were then washed three times in IP-buffer (20 mM HEPES (pH 7.5), 150 mM NaCl, 1 mM EDTA, and 1% Triton X-100) supplemented with complete EDTA-free protease inhibitors (#05056489001, Sigma Merck, Darmstadt, Germany) and phosSTOP (#04906837001, Roche Applied Science, Penzberg, Germany) before elution by boiling the samples in 2 × SDS loading buffer (0.75 M sucrose, 3.75% SDS, 31.25 mM Tris-HCl (pH 6.8), 0.1 mM EDTA (pH 7.5), 100 mM DTT, and 0.005% bromophenol blue). Non-relevant normal rat IgG (sc-2026, Santa Cruz, Dallas, TX, USA) or normal rabbit IgG (sc-2027, Santa Cruz, Dallas, TX, USA) antibodies were used as negative controls where indicated. Subsequent immunoblots were probed with anti-MLP (#R06991, GenScript, or sc-166930, Santa Cruz, as indicated) and anti-HAtag (#3724, Cell Signaling, Danvers, MA, USA) or anti-SDC4 (#429716, GenScript Biotech Corp, Piscataway, NJ, USA).

### 2.11. Immunoblotting

LV tissues were homogenized with TissueLyser (#85300, Qiagen Nordic, Venlo, The Netherlands) in ice-cold lysis buffer (20 mM Hepes (pH 7.5), 150 mM NaCl, 1 mM EDTA, and 0.5% Triton-X100) with an added complete EDTA-free protease inhibitor cocktail (#05056489001, Sigma Merck, Darmstadt, Germany) and PhosSTOP (#04906837001, Roche Applied Science, Penzberg, Germany). Primary rat cardiomyocytes and H9c2 cells were lysed in the same ice-cold lysis buffer or ice-cold RIPA buffer (#89900, Thermo Fisher Scientific, Waltham, MA, USA). The homogenates were centrifuged at 14000 RCF for 15 min at 4 °C, and the supernatants were stored at −20 °C or −80 °C. Protein concentrations were determined using a Micro BCA protein assay kit (#23235, Thermo Fisher Scientific, Waltham, MA, USA). An equal protein concentration was loaded per lane on 4–15 or 12% Criterion TGX precast gels (#5671084 and #5671044, Bio-Rad, Hercules, CA, USA) before being transferred onto PVDF membranes (#1704157, Bio-Rad, Hercules, CA, USA or #03010040001, Sigma Merck, Darmstadt, Germany) using the Trans-Blot Turbo system (#1704150, Bio-Rad, Hercules, CA, USA). The membranes were subsequently blocked in 1 × casein, 5% BSA, or 5% milk for one hour at room temperature before being incubated with primary antibodies overnight at 4 °C. After incubation, the membranes were washed in TBS-T (Tris-buffered saline with 1% Tween-20 (#1610781, Bio-Rad, Hercules, CA, USA)) for 20 min, followed by two 10 min washes. HRP-conjugated secondary antibodies were added for one hour at room temperature before another 20 min wash, followed by four 5 min washes in TBS-T. Using the ECL prime (#RPN2236, GE Healthcare, Arlington Heights, IL, USA), blots were developed, and the signal was thereafter detected with the Azure 600 Western blot imaging system (Azure Biosciences, Dublin, CA, USA). Membranes were stripped (#21603, Thermo Fisher Scientific, Waltham, MA, USA) for 5–10 min before reprobing. Total protein was detected using Revert 700 protein staining (#926-11021, LI-COR biosciences, Lincoln, NE, USA), and in the subcellular fractions, known compartment markers (GAPDH for cytoplasmic, Histone H3 for nuclear, NCX1 for membrane, and α-actinin for cytoskeleton) were used to verify the enrichment of the fractions.

### 2.12. Immunoblotting under Native Conditions

Where stated, the samples were run under native conditions. Here, the samples were diluted to the desired protein concentration (0.5 µg for recombinant proteins and 20 µg for rat cardiomyocyte subcellular fractions) in native sample buffer (#1610738, Bio-Rad, Hercules, CA, USA). The samples were not boiled prior to loading but were run with a standard SDS-containing running buffer (#1610772, Bio-Rad, Hercules, CA, USA) and transferred as described above.

### 2.13. Antibodies and Blocking Conditions

Anti-MLP (1:1000, 1 × Casein, sc-166930, Santa Cruz, Dallas, TX, USA), anti-MLP (1:1000, 1 × Casein, #R06991 and #R06990, GenScript Biotech Corp, Piscataway, NJ, USA), anti-syndecan-4 (1:1000, 1 × Casein, #429716, GenScript Biotech Corp, Piscataway, NJ, USA), anti-HA-tag (1:1000, 1 × BSA, #3724, Cell Signaling, Danvers, MA, USA), anti-NCX1 (1:1000, 1 × Casein, #3302, GenScript Biotech Corp, Piscataway, NJ, USA), anti-α-actinin (1:1000, 1 × Casein, ab68167, Abcam, Cambridge, UK), anti-GAPDH (1:500, 1 × Casein, sc-47724, Santa Cruz, Dallas, TX, USA), anti-Histone H3 (1:2000, 1 × Casein, #4499, Cell Signaling, Danvers, MA, USA), and anti-troponin T (1:1000, 1 × Casein, ab10214, Abcam, Cambridge, UK) were used. Secondary antibodies were anti-rabbit IgG HRP (N934V, Cytiva, Marlborough, MA, USA), anti-mouse IgG HRP (NA931V, Cytiva, Marlborough, MA, USA), and anti-goat IgG HRP (HAF109, R&D Systems, Minneapolis, MI, USA).

Where deemed necessary, a blocking peptide containing the epitope of the cytoplasmic syndecan-4 antibody (#429716, GenScript Biotech Corp, Piscataway, NJ, USA) was used to detect the specificity of bands. Here, the custom-made syndecan-4-blocking peptide (DLGKKPIYKKAPTN) was incubated with anti-syndecan-4 overnight at 4 °C prior to incubation of the membrane for 2 h at room temperature.

### 2.14. Immunofluorescence, Imaging and Processing

Baseline or differentiated H9c2 cells grown on glass coverslips or freshly isolated adult male LV cardiomyocytes were fixed in 4% paraformaldehyde for 10 min, quenched in 150 mM glycine for 10 min, and permeabilized with 0.5% Triton X-100 for 10 min at room temperature. Primary cardiomyocytes were additionally plated on glass-bottom dishes (No 1.5, Ø 14 mm, γ-irradiated, MatTek Corporation, Ashland, MA, USA) coated with laminin (#L2020, mouse, Sigma Merck, Darmstadt, Germany) and left to adhere for one hour at room temperature. Cells were then blocked in protein block (X0909, Dako, CA, USA) for 10 min at room temperature. For fluorescence labeling, cells were incubated with anti-MLP (1:50, sc-166930, Santa Cruz, Dallas, TX, USA), anti-MLP (1:50, #R06691, GenScript Biotech Corp, Piscataway, NJ, USA), anti-MLP (1:50, ab173301, Abcam, Cambridge, UK), anti-α-actinin (1:100, ab9465, Abcam, Cambridge, UK), anti-FLAG (1:50, F1804, Sigma Merck, Darmstadt, Germany), or anti-HA-tag (1:50, #3724, Cell Signaling technology, Danvers, MA, USA) overnight at 4 °C. After three 5 min washes, cells were incubated with anti-mouse Alexa Fluor 555 (1:200, #32727, Thermo Fisher Scientific, Waltham, MA, USA) and anti-rabbit Alexa Fluor 647 (1:200, #32733, Thermo Fisher Scientific, Waltham, MA, USA) for 1 h at room temperature. For the primary rat cardiomyocytes, DAPI 405 (0.1 mg/mL, #MBD0015, Sigma Merck, Darmstadt, Germany) was included in the secondary antibody step to visualize the nuclei. H9c2 cells were mounted with ProLong glass antifade mountant with NucBlue (P36983, Invitrogen, OR, USA). Both primary and secondary antibodies were diluted in a buffer containing 2% goat serum, 0.1% Triton X-100, and 0.02% NaN_3_ in PBS. As negative controls, freshly isolated primary rat cardiomyocytes and H9c2 cells were stained with primary or secondary antibodies only and DAPI. For imaging, cells were excited with the 555 and 647 nm laser lines of a ZEISS LSM 800 (Carl Zeiss AG, Jena, Germany) using the Airyscan mode and a plan-apo 63X 1.4 NA oil objective.

### 2.15. Statistics

All quantitative data are presented as the mean ± standard error of the mean. The normality of distribution was analyzed for all data using Shapiro–Wilk tests. Normally distributed data were tested with two-tailed unpaired *t*-tests, and non-normally distributed data with two-tailed Mann–Whitney U tests or Kruskal–Wallis with Dunn’s multiple comparisons (GraphPad Prism 9.4.1, La Jolla, CA, USA). Immunoblot quantifications were performed in FIJI 1.52p (NIH). Graph assembly and illustrations were made in Adobe Illustrator CS6 (Adobe Inc., San Jose, CA, USA).

## 3. Results

### 3.1. Syndecan-4 Binds to MLP in Nuclear-Enriched Fractions of Rat Primary Cardiomyocytes

**Figure 1:**
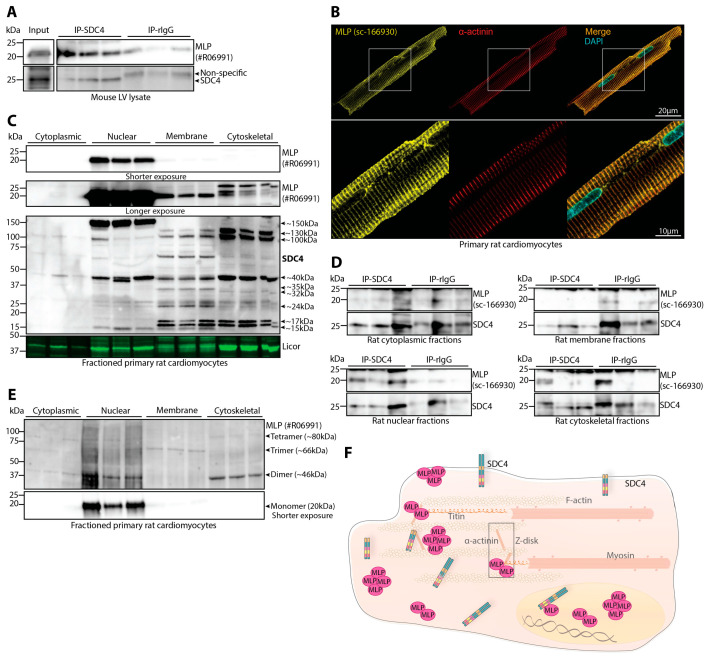
Syndecan-4 binds to MLP in nuclear-enriched fractions of rat primary cardiomyocytes. (**A**) Immunoprecipitation using anti-syndecan-4 (KY/8.2) in adult WT mouse LV lysates. Co-precipitation of MLP was detected with a custom-made antibody against the C-terminal region of MLP (specificity shown in Supplementary Figure S1A). Non-relevant rat IgG was used as a negative control (n = 3 mouse hearts). Syndecan-4 and non-specific precipitation are indicated by arrows on the right. Lysate input is shown on the left. (**B**) Representative immunofluorescence of MLP (yellow) and α-actinin (red) in freshly isolated adult rat primary cardiomyocytes. The cell nucleus was stained with DAPI (cyan). Negative control images are shown in Supplementary Figure S3A. (**C**) Immunoblot of MLP and syndecan-4 (SDC4) in enriched subcellular fractions of adult rat cardiomyocytes. A longer exposure image of the monomeric form of MLP is shown in the second panel down. Syndecan-4 positive bands are annotated with the approximate molecular weight on the right with arrows (specificity of bands are shown in Supplementary Figure S1F) (n = 3 rat hearts). Licor was used to show equal protein loading within fractions (20 µg). Subcellular compartment markers are shown in Supplementary Figure S1G. (**D**) Immunoprecipitation using anti-syndecan-4 (KY/8.2) in cytoplasmic-, nuclear-, membrane-, and cytoskeletal-enriched fractions of adult rat cardiomyocytes. Co-precipitation of MLP was detected with a custom-made antibody against the C-terminal end of MLP. Non-relevant rat IgG was used as a negative control (n = 3 rat hearts). (**E**) Immunoblotting of MLP under native conditions in cytoplasmic-, nuclear-, membrane-, and cytoskeletal-enriched fractions of adult rat primary cardiomyocytes (n = 3 rat hearts). A shorter exposure image of the monomeric form of MLP is shown in the lower panel. Interpreted oligomers and the approximate molecular weights are annotated on the right with arrows. (**F**) An illustration of the localization of syndecan-4 and MLP and their interaction in an adult rat cardiomyocyte, as identified in (**B**–**E**).

We have previously shown that syndecan-4 binds to MLP in rat left ventricular (LV) lysates [14]. To further verify this interaction also in mouse LV lysates, we performed immunoprecipitations of syndecan-4 using antibodies directed against the extracellular domain of syndecan-4 (epitope mapped in [14]). Consistent with our previous findings, immunoblotting with anti-MLP (custom made, #R06991, specificity in rat, mouse, and human is confirmed in Supplementary Figure S1A) showed that MLP co-precipitated more strongly with anti-syndecan-4 compared to a non-relevant rat antibody (rIgG) (Figure 1A). More pronounced co-precipitation was also observed in LV lysates from mice with cardiomyocyte-specific overexpression of syndecan-4 (Supplementary Figure S1B).

Furthermore, immunofluorescence confocal microscopy of primary rat cardiomyocytes using anti-MLP (sc-166930, specificity shown in Supplementary Figure S1C) revealed that MLP mainly localized to sarcomeric α-actinin-positive Z-discs (Figure 1B). Notably, the same staining pattern was obtained with two additional MLP antibodies (Supplementary Figure S1D–E). We also observed some MLP staining in the membrane, nucleus, and perhaps in the perinuclear space, where we have previously observed endogenous and HA-tagged syndecan-4 in bovine myocytes [37]. To our knowledge, there are currently no antibodies available against endogenous syndecan-4 that are suitable for immunofluorescence in mouse or rat cardiomyocytes, but previous work has observed a costameric localization [3].

To further determine the localization of MLP and syndecan-4, primary rat cardiomyocytes were subjected to subcellular fractionation before immunoblotting using standard denaturing conditions. The 20 kDa monomeric form of MLP was detected in the nuclear-, membrane-, and cytoskeletal-enriched fractions (Figure 1C, short and long exposures in the two upper most panels) but to a much higher extent in the nuclear-enriched fraction. Also, using a custom-made antibody against the cytoplasmic tail of syndecan-4 (epitope mapped in [2]), syndecan-4 (SDC4)-positive bands were observed in all fractions, although at varying sizes, which could represent different forms of the protein (Figure 1C, lower panel). The ~15–17 kDa and ~24 kDa bands observed in the nuclear-, membrane-, and cytoskeletal-enriched fractions are likely modified versions of the syndecan-4 cytoplasmic tail after shedding [10] and the core syndecan-4 protein as previously described [2,14,38], respectively. Additional syndecan-4-specific bands of different molecular weight (~32 kDa in the cytoskeletal, ~35kDa in the membrane, ~40 kDa in all fractions, ~100 kDa in the cytoplasm, membrane, and cytoskeleton, 130 kDa in the membrane and cytoskeleton, and ~150 kDa in nucleus and cytoskeleton) were also observed and were likely modified versions or SDS-resistant homo- or perhaps heterooligomers of syndecan-4 [39]. Except for the ~40 kDa band in the cytoskeletal fraction, all syndecan-4-positive bands appeared to be weaker or disappear in antibody-blocking experiments, suggesting the bands to be of syndecan-4 origin (Supplementary Figure S1F). Enrichment of the fractions was confirmed by subcellular compartment markers (Supplementary Figure S1G).

To explore which subcellular fraction the syndecan-4–MLP interaction occurs in, we performed immunoprecipitations in each fraction. MLP co-precipitated with syndecan-4 (SDC4) in the nuclear-enriched fraction only, compared to the non-relevant rat IgG (Figure 1D, bottom left panel). MLP did not co-precipitate with syndecan-4 in the other enriched fractions.

Finally, as the localization of MLP in neonatal cardiomyocytes has previously been found to depend on its oligomeric state [29], we immunoblotted for MLP in the adult rat cardiomyocyte subcellular-enriched fractions under native conditions. For the detection of oligomeric MLP, a custom-made antibody against the C-terminal end of the MLP was produced (IGFGGLTQQVEKKE), as previous studies have found that this epitope is able to detect MLP oligomers [29] (epitope mapped in Supplementary Figure S1A). Consistent with the results from neonatal cardiomyocytes, the 20 kDa monomeric form of MLP was observed mainly in the nuclear-enriched fraction (Figure 1E, lower panel). In neonatal rat cardiomyocytes and human hearts, oligomeric MLP has previously been reported at the molecular weights of ~46 kDa, ~66 kDa, and ~80 kDa for the dimer, trimer, and tetramer, respectively [29]. We observed similar molecular weights of MLP in adult rat cardiomyocytes, where the dimeric form at ~46 kDa was detected mainly in the cytoskeletal- and nuclear-enriched fractions and faintly in the cytoplasmic fraction (Figure 1E, upper panel). Trimeric MLP at ~66 kDa was mainly detected in the membrane fraction. Lastly, tetrameric MLP at ~80 kDa was weakly detected in the cytoplasmic-, nuclear-, and cytoskeletal-enriched fractions.

Taken together, MLP mainly localized to the α-actinin-positive Z-discs but also to the membrane, nucleus, and perhaps perinuclear regions in adult rat cardiomyocytes and interacted with syndecan-4 in the nuclear region, as illustrated in Figure 1F. MLP monomers, dimers, trimers, and tetramers were all observed in the adult cardiomyocyte, where the MLP monomer was highly enriched in nuclear fractions.

### 3.2. Syndecan-4 Binds to Multiple Sites of MLP

**Figure 2:**
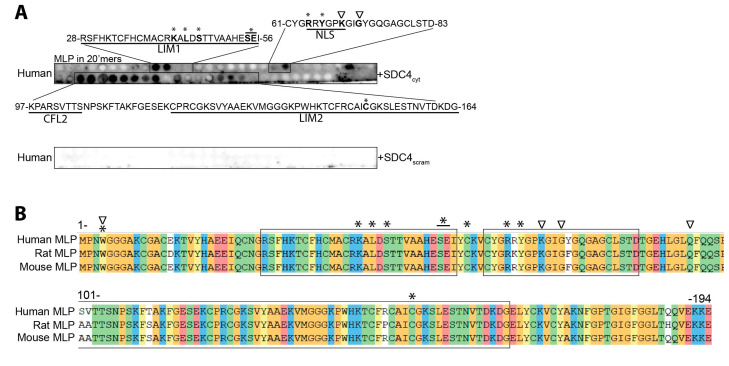

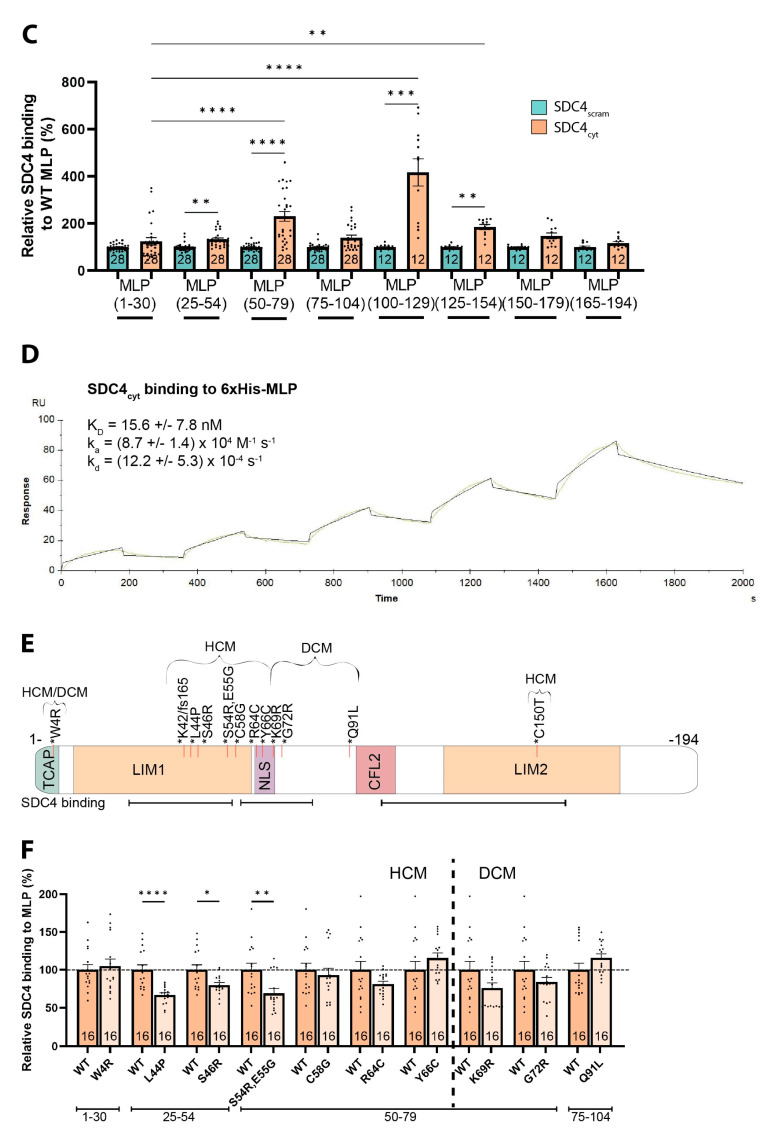
Syndecan-4 binds to multiple sites in MLP. (**A**) Human MLP-WT was synthesized as 20’mer overlapping peptides with 3 amino acids offset on a membrane and overlaid with a biotinylated SDC4_cyt_ peptide (upper membrane). An identical membrane overlayed with a biotinylated SDC4_scram_ peptide was used as a negative control (lower membrane) (n = 3). Boxed regions indicate the strongest syndecan-4 binding sites in MLP-WT, annotated with corresponding amino acid sequences. Specific domains are underlined and known human mutations in MLP are in bold and annotated with a star (HCM-associated) or triangle (DCM-associated). (**B**) Protein sequence alignment of human, rat, and mouse MLP (color by chemistry) shows almost complete sequence similarity across species. SDC4_cyt_ binding domains identified in (**A**) are annotated with boxes, and known human MLP mutations are annotated with a star (HCM-associated) or triangle (DCM-associated). (**C**) An ELISA-based assay coated with overlapping MLP-WT peptides (annotated beneath the graph) overlayed with SDC4_cyt_ or SDC4_scram_ peptides (n = 12–28). All values are presented as mean percentages ± SEM where SDC4_cyt_ was normalized to a corresponding SDC4_scram_ control peptide (set to 100%). (**D**) SPR analyses of syndecan-4 and MLP were conducted by immobilizing biotin-ahx-SDC4_cyt_ on an SA chip. The response was measured while injecting a range of concentrations of recombinant 6xHis-MLP-WT. The green curve shows the experimental data, and the black shows the mathematically modeled fit (n = 4). (**E**) An illustration of known MLP binding domains and mutations known to associate with the HCM or DCM phenotype in humans, located mainly in the LIM1 and NLS regions of MLP. The syndecan-4 binding domains identified in (**A**,**C**) are annotated beneath the illustration. (**F**) An ELISA-based assay coated with 30 amino acid-long MLP-WT or mutated MLP peptides overlayed with SDC4_cyt_ or SDC4_scram_ (n = 16). Values are presented as mean percentages ± SEM where SDC4_cyt_ peptides were normalized to the corresponding SDC4_scram_ peptides. MLP mutated peptides were then related to the MLP-WT peptide (set to 100%, dashed line). Differences between SDC4_cyt_ and SDC4_scram_, and MLP-WT and MLP mutations were tested with a Kruskal–Wallis test with Dunn’s multiple comparisons (**C**) and Mann–Whitney U tests (**F**), respectively, due to non-normal distribution analyzed by Shapiro–Wilk testing (* *p* < 0.05, ** *p* < 0.01, *** *p* < 0.001, **** *p* < 0.0001).

We next aimed to identify the syndecan-4-MLP-binding domain(s). A biotinylated peptide covering the cytoplasmic domain of syndecan-4 (SDC4_cyt_) was overlaid onto 20’mer overlapping peptides covering the full length of human MLP. Biotinylated SDC4_cyt_ bound to three sequences on human MLP; 28-RSFHKTCFHCMACRKALDSTTVAAHESEI-56, located in the LIM1 domain, 61-CYG**RRYGPK**GIGYGQGAGCLSTD-83, covering the nuclear localization signal (NLS) of MLP (in bold) [40] and 97-KPARSVTTSNPSKFTAKFGESEKCPRCGKSVYAAEKVMGGGKPWHKTCFRCAICGKSLESTNVTDKDG-164, partially covering the cofilin-2 (CFL2) and LIM2 domains (Figure 2A, upper panel). Importantly, control scrambled peptides, biotinylated SDC4_scram_, did not recognize any human MLP sequence (Figure 2A, lower panel). The protein sequence alignment of mouse, rat, and human MLP showed that the amino acids within the three SDC4_cyt_ binding sites were almost identical across the three species (Figure 2B). In an ELISA-based assay, using wells coated with 30 amino acid-long overlapping MLP peptides, biotin-SDC4_cyt_ binding was confirmed with four MLP peptides covering the same sequences, namely amino acids 25–54, 50–79, 100–129, and 125–154 (Figure 2C).

The binding of SDC4_cyt_ and MLP was further investigated with surface plasmon resonance (SPR), where a biotinylated SDC4_cyt_ peptide was immobilized on an SA chip, and a serial dilution of recombinant 6xHis-MLP-WT protein was injected over the chip. The results were analyzed by fitting with a 1:1 interaction model (Langmuir). The dissociation equilibrium constant (k_D_) was 15.6 ± 7.8 nM, with an association constant (k_a_) of (8.7 ± 1.4) × 10^4^ M^−1^ s^−1^ and a dissociation rate constant (k_d_) of (12.2 ± 5.3) × 10^−4^ s^−1^, indicating a strong interaction (Figure 2D). Although the software was able to mathematically fit the interaction, there was an indication that MLP oligomerization influenced the interaction, observed by a possible saturation at the first curve, potentially being monomeric MLP, followed by an increase in slope angle at the remaining protein injections, potentially influenced by oligomeric MLP binding.

Altogether, our data indicated that syndecan-4 binds to distinct sequences of MLP in vitro, covering the LIM1 domain, the NLS, part of the CFL2 binding domain, and part of the LIM2 domain (illustrated beneath Figure 2E).

### 3.3. Syndecan-4 Has an Altered Binding to Cardiomyopathy-Associated MLP Mutations

Multiple human mutations in MLP are associated with hypertrophic cardiomyopathy (HCM) or dilated cardiomyopathy (DCM) (illustrated in Figure 2E). Interestingly, many of the mutation sites are located in the syndecan-4–MLP interacting domains. To test whether syndecan-4 binding to MLP was affected by any of the HCM- or DCM-associated mutations, we used an ELISA-based assay, where wells were coated with WT or mutated MLP peptide sequences. Our results showed that the biotinylated SDC4_cyt_ bound significantly weaker when MLP was mutated at L44P, S46R, or S54R, E55G (Figure 2F). Additionally, there was a tendency of reduced binding to several other MLP mutations, including R64C and the two DCM-mutations K69R and G72R. As expected, the disease-modifying W4R mutation did not alter the binding of SDC4_cyt_, consistent with the lack of syndecan-4 binding detected in this region.

### 3.4. Analysis of Syndecan-4 and MLP, and Their Interaction, in H9c2 Cells at Baseline and after Differentiation

**Figure 3:**
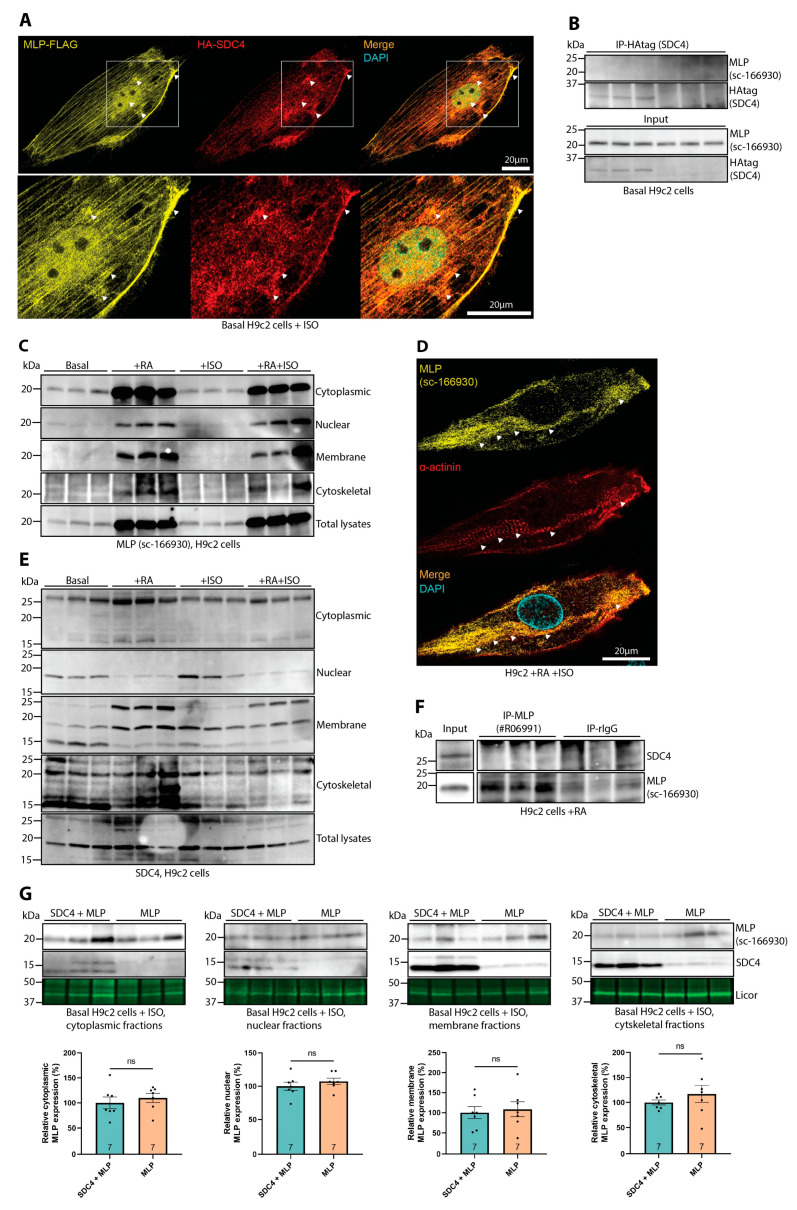
Syndecan-4 does not bind directly to MLP in H9c2 cells. (**A**) Representative immunofluorescence of MLP-FLAG (yellow) and HA-syndecan-4 (red) in H9c2 cells stimulated with ISO. The cell nucleus was stained with DAPI (cyan). Negative control images are shown in Supplementary Figure S3B. (**B**) H9c2 cells were transfected with HA-SDC4 and untagged MLP at basal conditions, harvested in lysis buffer, and immunoprecipitated using anti-HA (SDC4). A possible co-precipitation of MLP was analyzed with an antibody against MLP. Lysate inputs are given in the two lowermost panels (n = 6). (**C**) Immunoblot analysis of MLP in cytoplasmic-, nuclear-, membrane-, and cytoskeletal-enriched H9c2 cell fractions at basal conditions +/− ISO, or in the presence of retinoic acid (+RA) +/− ISO (n = 6). (**D**) Immunofluorescence of endogenous MLP (yellow) and sarcomeric α-actinin (red) in H9c2 cells differentiated in the presence of retinoic acid (+RA) and treated with 25 µM ISO. The cell nucleus was stained with DAPI (cyan). (**E**) Immunoblot analysis of syndecan-4 in cytoplasmic-, nuclear-, membrane-, and cytoskeletal-enriched H9c2 cells at basal conditions +/− ISO or in the presence of RA +/− ISO (n = 6) (specificity of bands is shown in Supplementary Figure S2E). (**F**) Lysates from H9c2 cells differentiated with RA were harvested in lysis buffer and subjected to immunoprecipitation using anti-MLP (antibody epitope mapped to aa 169–194 in the extreme C-terminus, Supplementary Figure S1A) and subsequent immunoblotting of SDC4 (n = 6). Lysate input is given in the left most panels. (**G**) H9c2 cells transfected with untagged MLP +/− SDC4 were subjected to subcellular fractionation, and syndecan-4- and MLP levels were assessed by immunoblotting. The ~15 kDa shed syndecan-4 fragment was used as a control for overexpression. Subcellular compartment markers were used to show protein loading. Densitometry levels were subsequently quantified (n = 7–13). All values in (**G**) are presented as mean percentages ± SEM, normalized to subcellular compartment markers, and MLP was thereafter related to SDC4 + MLP. Differences in MLP protein levels were tested with Mann–Whitney U tests due to non-normal distribution analyzed by Shapiro–Wilk testing (ns, not significant).

To better understand the underlying molecular mechanism of MLP-associated cardiomyopathy and the potential involvement of syndecan-4, we tested whether the H9c2 rat ventricular cardiomyoblast cell line could be used as a model [41,42]. Immunofluorescence confocal microscopy of H9c2 cells transfected with a C-terminally FLAG-tagged MLP (MLP-FLAG) and HA-tagged syndecan-4 (HA-SDC4) at baseline conditions revealed that these proteins had some overlap at the cell membrane, around and in the nucleus, and throughout cell striations (Figure 3A). However, immunoprecipitation of HA-SDC4 with the HA-tag antibody revealed no co-precipitation of MLP (Figure 3B, upper panel; input is shown in the two lowermost panels). To exclude the possibility of the tags interfering with the protein folding or interaction, we also performed immunoprecipitation of the endogenous proteins, but no specific binding of the two proteins was detected (Supplementary Figure S2A).

Since immature H9c2 cardiomyoblasts lack the morphology and contractile apparatus of primary adult cardiomyocytes, as seen by the lack of structural organization of sarcomeric α-actinin immunofluorescence staining (Supplementary Figure S2B), we differentiated the H9c2 cells into cardiomyocyte-resembling cells. Differentiation of H9c2 cardiomyoblasts was conducted by adding 10 nM all-trans retinoic acid (RA) daily for 5 days in reduced media serum concentrations (1%) [43]. Since we and others have previously found the nuclear translocation of MLP to be dependent on phenylephrine or isoprenaline (ISO) treatment, contractility, or mechanical strain [14,40], some H9c2 cells were also stimulated with the β-adrenergic agonist ISO (25 µM) where indicated, 24 h prior to harvest. At baseline conditions, MLP was mainly detected in the cytoplasmic- and nuclear-enriched fractions, whereas upon differentiation (+RA), increased MLP levels were observed in all four fractions, including the membrane- and cytoskeletal-enriched fractions (Figure 3C). Equal loading was verified by Ponceau S staining (Supplementary Figure S2C), and the enrichment of fractions was analyzed by GAPDH, Histone H3, NCX1, and α-actinin (Supplementary Figure S2D). Consistently, immunofluorescence confocal microscopy revealed that MLP started to localize to α-actinin-positive structures that morphologically resemble Z-discs (Figure 3D, white arrows). The ISO treatment did not seem to have a large effect on MLP localization (Figure 3C).

Interestingly, differentiation (+RA) induced a shift in syndecan-4 subcellular localization. The largest effects of differentiation and ISO treatment on the syndecan-4-positive bands were seen between 15–25 kDa. After differentiation, syndecan-4 levels were decreased in nuclear-enriched fractions, and increased in the membrane. (Figure 3E). The specificity of the syndecan-4-positive bands was confirmed by antibody-blocking experiments (Supplementary Figure S2E). Despite the alterations of syndecan-4 and MLP in differentiated H9c2 cells, immunoprecipitation revealed that the two proteins still did not co-precipitate (Figure 3F). Successful differentiation was confirmed by upregulation of cardiomyocyte markers, like troponin T and sarcomeric α-actinin (Supplementary Figure S2F). To test if the interaction was present in more advanced cells, we performed immunoprecipitation of syndecan-4 in HL-1 atrial cardiomyocytes and neonatal rat cardiomyocytes. While MLP was not co-precipitated in HL-1 cells (Supplementary Figure S2G), MLP was co-precipitated in neonatal rat cardiomyocytes (Supplementary Figure S2H).

We have previously found that the lack of endogenous syndecan-4 decreases the levels of nuclear-located endogenous MLP protein levels in the LVs of mice, and conversely, viral overexpression of syndecan-4 increases the levels of nuclear-located endogenous MLP in H9c2 cells [14]. To test whether overexpression of syndecan-4 could affect the subcellular localization of MLP in H9c2 cells, we transiently transfected H9c2 cells with MLP-WT +/− syndecan-4 and stimulated them with ISO (25 µM). The cells were fractioned and subsequently analyzed by immunoblotting for syndecan-4 and MLP. We used the ~15 kDa shed syndecan-4 fragment as a positive control for overexpression, as has been done by others [2,10]. Under these conditions, there was no significant difference in the localization of MLP in any of the fractions, regardless of the presence of syndecan-4 (Figure 3G).

Taken together, our results indicated that exogenously expressed syndecan-4 and MLP might co-localize in the membrane, around and in the nucleus, and in striations of the H9c2 cells at baseline conditions; however, no interaction was detected between either exogenous or endogenous MLP and syndecan-4. Although the differentiation of H9c2 cells increased the levels of endogenous MLP in all subcellular fractions and altered syndecan-4 localization, no syndecan-4–MLP interaction was observed. Lastly, exogenous syndecan-4 expression had no effect on MLP localization in H9c2 cells.

### 3.5. The Effect of Human MLP Mutations on Its Subcellular Localization in H9c2 Cells

**Figure 4:**
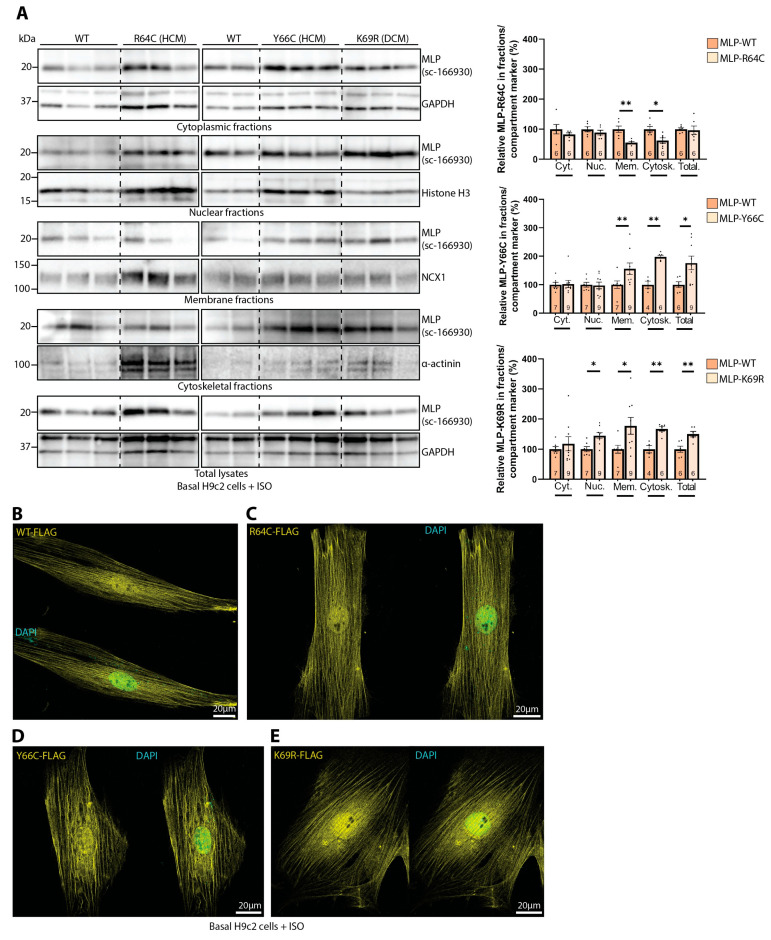
The effect of MLP mutations on subcellular localization. (**A**) H9c2 cells transfected with WT or mutated MLP and stimulated with 25 µM isoprenaline (ISO) were subjected to subcellular fractionation and subsequent immunoblotting for MLP (right panels) (dotted lines indicate representative bands are a montage from the same exposed blot). Total lysates were harvested in RIPA buffer. Densitometry quantifications shown on the right (n = 4–9) are presented as mean percentages ± SEM where MLP levels were normalized to respective subcellular compartment markers before mutated MLP was related to MLP-WT. Differences were tested with Mann–Whitney U tests due to non-normal distribution analyzed by Shapiro–Wilk testing (* *p* < 0.05, ** *p* < 0.01). Immunofluorescence of (**B**) MLP-WT-FLAG, (**C**) R64C-FLAG, (**D**) Y66C-FLAG, and (**E**) K69R-FLAG (yellow) transfected into H9c2 cells and stimulated with 25 µM ISO. The cell nuclei were stained with DAPI (cyan). Negative control images are shown in Supplementary Figure S3B.

Next, we tested whether HCM- or DCM-associated MLP mutations could have any effect on the subcellular localization of MLP. Since mutations located in the LIM1 domain have previously been found to be prone to degradation due to destabilized protein folding [44], we chose the human mutations located in the NLS, R64C, and Y66C (HCM) as well as K69R (DCM) for further analyses. H9c2 cells were transfected with mutated MLP (untagged) and stimulated with 25 µM ISO for 24 h prior to harvest, as we have previously shown that ISO contributes to the nuclear translocation of MLP in H9c2 cells [14]. Subcellular fractions and total RIPA lysates were immunoblotted for MLP (Figure 4A, left panels) using an antibody recognizing an epitope outside the mutated region (Supplementary Figure S1C, epitope mapped to amino acids 73–104). Subsequent densitometry quantification revealed that the HCM-associated mutation MLP-R64C was decreased in the membrane- and cytoskeletal-enriched fractions (Figure 4A, right and left panels, MLP-R64C vs. MLP-WT). In contrast, expression of the MLP-Y66C (HCM) and MLP-K69R (DCM) variants was increased in the membrane- and cytoskeleton-enriched fractions, as well as in the total lysates, compared to MLP-WT (Figure 4A, right and left panels, MLP-Y66C and -K69R vs. MLP-WT). MLP-K69R also had higher levels of MLP in the nuclear-enriched fraction (Figure 4A, right and left panels, MLP-K69R vs. MLP-WT). Interestingly, the DCM-associated MLP-K69R mutation has been reported to have an increased perinuclear localization in C2C12 cells compared to a widespread localization of MLP-WT [34]. In the H9c2 cells transfected with FLAG-tagged MLP-WT or mutated MLP and stimulated with ISO 24 h prior to fixation, MLP-WT was mainly localized in the nuclear region and striations throughout the cell (Figure 4B). The two HCM-associated mutations, MLP-R64C and MLP-Y66C, and the DCM-associated mutation, MLP-K69R, also localized to the nuclear region and cell striations, much like MLP-WT (Figure 4C–E). Consistent with the fraction analysis, there was also a tendency for the increased nuclear region staining of MLP-K69R.

### 3.6. Human Mutations in MLP Alter Its Oligomerization Potential

The functional effects of MLP have previously been linked to its oligomeric state [29]. We, therefore, hypothesized that human mutations in MLP could alter its oligomeric potential.

**Figure 5:**
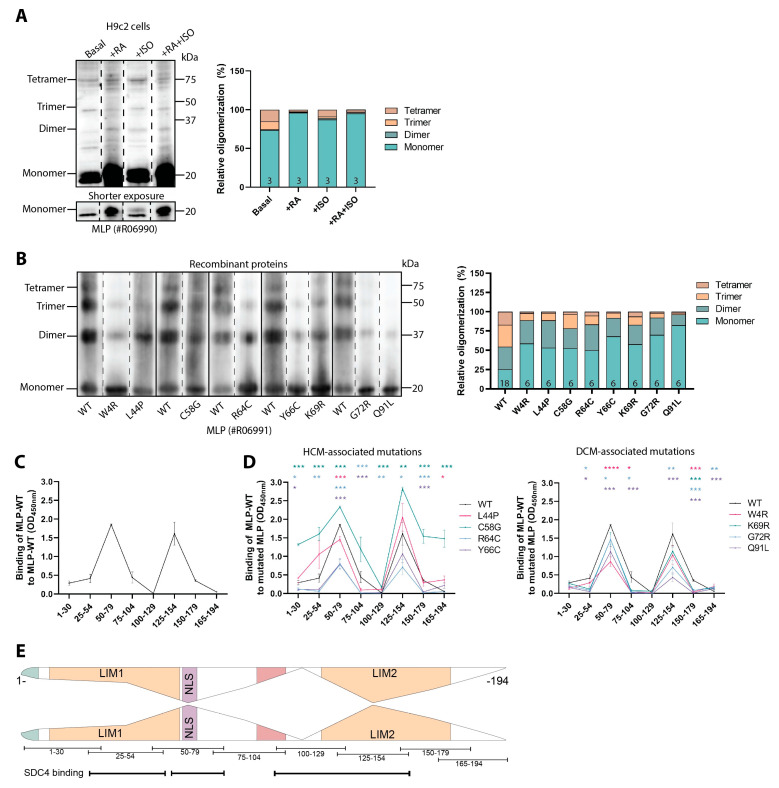
Mutations in MLP alter its oligomerization potential. (**A**) H9c2 cells treated with +/− retinoic acid (RA) +/− isoprenaline (ISO) and harvested in lysis buffer were subjected to immunoblotting of MLP under native conditions (left panel, dotted line indicates representative lanes are a montage from the same exposed blot). Oligomers of MLP-WT are shown as a percentage of the total MLP detected by densitometry quantification (right panel, n = 3). (**B**) Recombinant WT or mutated 6xHis-MLP proteins were analyzed by native immunoblotting to measure oligomerization levels (left panel, dotted line indicates representative lanes are a montage from the same exposed blot). Monomers and oligomers for WT and each MLP mutation are shown as a percentage of the total MLP level detected by densitometry quantification (right panel, n = 6–18). Percentage values ± SEM and statistical analyses are given in Supplementary Table S1. (**C**) An ELISA-based assay coated with full-length recombinant 6xHis-MLP-WT protein, incubated with biotinylated overlapping MLP-WT peptides as potential binding partners (n = 8). (**D**) An ELISA-based assay coated with eight recombinant 6xHis-MLP mutated proteins, incubated with biotinylated overlapping MLP-WT peptides as potential binding partners (n = 8). Signals from wells coated with PBS only were subtracted from experimental wells (**C**,**D**). Binding of MLP-WT peptides to recombinant 6xHis MLP-WT or MLP mutated proteins are shown as mean ± SEM (**C**,**D**). Differences between MLP-WT and mutated MLP for each peptide sequence were tested by Mann–Whitney U tests due to non-normal distribution analyzed by Shapiro–Wilk testing (* *p* < 0.05, ** *p* < 0.01, *** *p* < 0.001, **** *p* < 0.0001) (**D**). (**E**) An illustration of MLP self-association results obtained from (**C**). Peptide sequences and syndecan-4 binding sites identified in Figure 2 are annotated beneath the illustration. Illustrative self-association assumes symmetrical 1:1 binding, which we have not confirmed.

To characterize the oligomeric state of endogenous MLP in H9c2 cells, total lysates were immunoblotted for MLP under native conditions. In all conditions, the H9c2 cells exhibited mainly monomeric MLP, with a small fraction of total MLP also being oligomeric (Figure 5A). Differentiation and ISO stimulation increased the levels of monomeric MLP; however, minimal changes in the levels of oligomeric MLP were detected (Figure 5A). Due to such large quantities of MLP in H9c2 cells being monomeric upon differentiation and differentiated cells becoming quite resistant to transient liposomal transfection, a further oligomerization investigation was performed with the recombinant proteins and peptides.

Immunoblotting under native conditions of recombinant 6xHis-MLP-WT and mutated proteins with the custom-made C-terminal MLP antibody (#R06991) revealed that MLP-WT had an approximate 25% distribution of monomers and each oligomer. All mutations increased the monomeric form of MLP at the expense of the trimeric and tetrameric oligomers (Figure 5B, left and right panel and Supplementary Table S1).

Various studies from others have suggested both the N- and C-terminal ends to be involved in oligomerization [29,45]. To more precisely map the sites of self-association in MLP, an ELISA-based screening method was used. Full-length recombinant 6xHis-MLP-WT protein was coated into wells and overlayed with biotinylated overlapping MLP-WT peptides, together representing the full-length MLP sequence. Our results indicated that several regions of MLP were involved in self-association (Figure 5C). Interestingly, the strongest binding was seen at amino acids (aa) 50–79, covering the end of LIM1 and the nuclear localization signal (NLS), and aa 125–154, covering the middle of the LIM2 domain, suggesting these regions are important sites of oligomerization. Minimal binding was observed at aa 100–129, representing the end of the cofilin-2 binding domain and the start of LIM2, and aa 165–194, representing the end of the LIM2 domain. Furthermore, to test whether mutations could affect self-association, wells were instead coated with mutated recombinant 6xHis-MLP proteins and overlayed with the biotinylated MLP-WT peptides, mimicking a heterozygous interaction. Compared to the WT recombinant protein, MLP peptides aa 50–79 and 125–154 bound weaker to most of the mutated MLP recombinant proteins, except for the HCM-associated L44P (at aa 125–154) and C58G mutations (at aa 50–79 and 125–254) (Figure 5D, left and right panel). A larger increase in N- and C-terminal binding was also observed for C58G.

Altogether, our data indicate that the majority of endogenous MLP in H9c2 cells was monomeric and that recombinant MLP showed reduced oligomerization when mutated. MLP self-association was mapped to the end of LIM1, NLS, and the middle of the LIM2 domains (illustrated in Figure 5E with the assumption of symmetrical 1:1 binding). Self-association was, for the most part, reduced when MLP was mutated.

## 4. Discussion

In this study, we have investigated syndecan-4 and MLP and their interaction in primary rat adult cardiomyocytes and H9c2 cells. We have previously shown that syndecan-4 and MLP co-precipitate in total rat LV lysates [14]. Here, we show that this interaction is specific to the nuclear-enriched fraction of isolated adult cardiomyocytes. In primary adult rat cardiomyocytes, MLP co-localized to α-actinin-positive Z-discs, and the oligomeric-dependent subcellular localization was somewhat different from that which others have previously described in neonatal cardiac myocytes. Our in vitro work suggests that syndecan-4 binds to MLP at three distinct domains, covering part of the LIM1 and LIM2 domains, but also the nuclear localization signal (NLS). Additionally, syndecan-4 appeared to bind weaker to hereditary HCM-associated mutations L44P, S46R, and S54R, E55G in vitro. Despite a thorough investigation into the rat cardiomyoblast H9c2 cell line, the syndecan-4–MLP interaction was not present. However, independent of syndecan-4, transfection of mutated and WT MLP yielded an altered subcellular localization of mutated MLP compared to the WT. Furthermore, mutations in MLP also induced changes to the oligomeric state of recombinant MLP proteins, where all mutations had a higher degree of monomeric at the expense of trimeric and tetrameric MLP. Lastly, our data suggests that MLP self-association is dependent on two regions, the first spanning the end of LIM1 and the NLS and the second covering the middle of the LIM2 domain, which is weakened when MLP is mutated.

To better understand the underlying mechanism of MLP-associated cardiomyopathy and the potential involvement of syndecan-4, we employed adult cardiomyocytes to determine the subcellular fraction in which this interaction occurs. Interestingly, we only detected the syndecan-4–MLP interaction in the nuclear compartment. We have previously found that the syndecan-4 knockout (KO) mouse possesses less MLP in nuclear fractions [14], suggesting that syndecan-4 is involved in the nuclear translocation of MLP. Our present data suggests that syndecan-4 and MLP, although co-localizing in the other subcellular compartments, do not bind before having entered the nuclear area, at least in isolated cardiomyocytes. However, compared to others, we observed little MLP in the cytoplasmic-enriched subcellular fraction [31]. It should be noted that the isolation procedure may have stressed the cells, potentially having led to the nuclear translocation of MLP. We can, therefore, not disregard the possibility that syndecan-4 and MLP bind in other subcellular fractions in non-isolated cardiomyocytes in tissue. An additional possibility is that syndecan-4 requires modifications to facilitate its binding to MLP in other subcellular fractions. Dephosphorylation of serine 179 of syndecan-4 upon pressure overload has, for example, been found to increase its binding to calcineurin, activating the nuclear factor of activated T-cell (NFAT) pro-hypertrophic signaling [2]. It is possible the increased binding between syndecan-4 and MLP we have previously observed in the pressure-overloaded heart is due to similar changes in the phosphorylation level [14]. This is an interesting topic that will be investigated in future studies. Both MLP and syndecan-4 possess a NLS sequence, of which the NLS motif RMKKK in syndecan-4 seems to be conserved across the syndecan family [37,40,46]. Syndecan-4 may be involved in stabilizing MLP in the nucleus; hence, its loss results in lower levels of MLP. Although MLP does not contain a DNA binding domain, it has been suggested to act as a transcriptional activator through its physical interaction with basic helix-loop-helix transcription factors, such as myogenin, enhancing skeletal myogenesis [47].

The nuclear localization of different forms of syndecan-4 is also an interesting observation. Nuclear localization of syndecan-1 has also been demonstrated by others in mesothelioma cells, hepatocytes, and corneal fibroblasts, with suggested functions in the regulation of cell proliferation, synthesis of RNA, and splicing [46,48,49]. Additionally, we have previously also observed the co-localization of syndecan-4 and Lamin A in bovine muscle cells [37]. The exact role of syndecan-4 and its different forms in the nuclear region is an interesting area of further study.

As has been reported in neonatal rat cardiomyocytes, our data suggest that MLP oligomerization is also associated with a specific subcellular localization in adult rat cardiomyocytes [29]. We found that MLP monomers were mainly present in nuclear-enriched fractions, whereas dimers were present in both the nuclear and cytoskeletal compartments and less in the cytoplasm. MLP trimers were mainly observed in the membrane, and tetramers were detected weakly in the cytoplasm and nuclear fractions. In previous work using neonatal cardiac myocytes, only monomeric MLP was found in the nucleus, while dimeric and trimeric were found in the membrane, and only tetrameric MLP was detected in the cytoskeleton [29]. To our knowledge, oligomerization has not been investigated in adult cardiomyocytes before. The differences observed are likely due to the developmental stage of the cardiomyocytes used, where the function, and therefore subcellular localization of MLP, is different. Neonatal cardiac myocytes are subject to maturation through processes such as myofibril assembly through t-tubule development and sarcomere organization, an increase in cell size, and the development of contractile properties [50]. During these processes, it could be hypothesized that MLP binds to different protein partners at different subcellular locations, in turn determining its oligomeric state.

The form of MLP to which syndecan-4 preferentially binds to is currently unknown. Since the two proteins bind in the nuclear region, it is likely that the interaction involves monomeric or dimeric MLP, which we detected in this compartment with immunoblotting under native conditions. Our in vitro analysis of the interaction suggests a strong binding of syndecan-4 to MLP at three sites, including part of the LIM1 domain, the NLS, part of CFL2, and part of the LIM2 domain. Whether these sites reflect the true binding domains of syndecan-4 to the tertiary MLP protein cannot be elucidated by the use of these methods. Since the use of short peptides may disrupt the folding, especially in regard to the zinc-finger containing LIM domains, these results should be interpreted with caution. However, the SPR analysis of the interaction suggests that syndecan-4 may be able to bind both the monomeric and oligomeric forms of MLP. As both MLP and syndecan-4 can oligomerize, we cannot exclude that the immobilized syndecan-4 or MLP are oligomeric forms or that syndecan-4 oligomerization is necessary for its binding to MLP. Syndecan-4 dimerization has, for example, been found to be needed for the activation of its well-described binding partner protein kinase C α (PKCα), which has been implicated in focal adhesion formation, migration, and adhesion [51,52].

What happens to the interactions between MLP and its protein-binding partners when MLP is mutated has not been previously investigated in detail. We observed lower levels of binding of syndecan-4 to several of the HCM-associated MLP variants, namely L44P, S46R and S54R, E55G. Similarly, the direct interaction that has been found between the LIM1 and NLS domains of MLP and α-actinin has been found to be reduced when MLP is mutated at C58G and K69R, likely contributing to the cardiomyopathy phenotypes in these patients [34,53,54]. Whether the L44P, S46R and S54R, E55G mutations lead to weakened binding between syndecan-4 and MLP in the nuclear region of cardiomyocytes in vivo or perhaps reduce the nuclear entry of MLP altogether remains to be investigated.

To further examine the interaction between syndecan-4 and MLP in cells, we tested whether the H9c2 rat cardiomyoblast cell line could be used as a model system [42]. Despite some co-localization in and around the nucleus and in cell striations, the interaction between syndecan-4 and MLP was not detectable in H9c2 cells at baseline conditions. It could be postulated that at the immature cardiomyoblast stage, MLP has not yet been structurally placed at the subcellular localization where it will exert its effect at a mature stage, and therefore, the syndecan-4–MLP interaction is not yet present. This hypothesis is also supported by observations in the MLP KO mouse, which survives the embryonic period but suffers post-natal lethality, linked to a deficient terminal differentiation of cardiomyocyte cytoarchitecture [18]. Similarly, in human embryonic stem cells lacking MLP, loss of MLP does not affect the initial differentiation into cardiomyocytes, while more differentiated cells display a typical HCM phenotype [55]. To test whether an augmented differentiation of H9c2 cells into cardiomyocyte-resembling cells improved MLP and syndecan-4 co-localization, we treated the cells with all-trans-retinoic acid and reduced the serum concentrations [56]. As expected, the better-differentiated cells started to express cardiac markers such as cardiac troponin T. Immunofluorescence imaging also showed an increase in sarcomeric α-actinin organization throughout the cell and indications of its co-localization with MLP. The binding between endogenous syndecan-4 and MLP was, however, still not present in these cells. Additionally, MLP was found not to co-immunoprecipitate with syndecan-4 in the HL-1 atrial cardiomyocytes. Together, these data indicate that the syndecan-4–MLP interaction is likely only present in primary cardiomyocytes since we observed this binding in rat nuclear-enriched subcellular fractions, mouse LV lysates, cardiomyocyte-specific syndecan-4 overexpressing mouse LV lysates, and neonatal rat cardiomyocytes. These findings indicate that the syndecan-4–MLP interaction may require specific types of post-translational modifications to these proteins or associated partners such as titin-TCAP [22]. These post-translational modifications may only be present in primary cells.

To determine if any effect of syndecan-4 overexpression could be observed on the MLP subcellular localization in H9c2 cells, they were transiently transfected with MLP in the presence or absence of syndecan-4. Under these conditions, we did not see any effects of syndecan-4 on MLP subcellular localization. We have previously found that the overexpression of syndecan-4 increases the nuclear-located MLP in H9c2 cells; however, this was with the viral overexpression of syndecan-4, investigating endogenous MLP [14], perhaps yielding a higher level of overexpression.

Independently of syndecan-4, we further investigated the subcellular localization of MLP-WT and MLP mutations in the NLS signal, such as R64C, Y66C, and K69R in H9c2 cells. None of the three mutations were affected by proteolytic degradation, at least in the H9c2 cells. Both MLP-Y66C and MLP-K69R had increased levels of MLP in the membrane- and cytoskeleton-enriched fractions. We also observed an increase in the nuclear localization of K69R by subcellular fractionation and confocal microscopy. In line with our data, an increased perinuclear localization of K69R has also been observed in C2C12 cells [34]. In contrast, mutations such as C58G, which are located in the LIM1 domain, have been found to have a gene-dose-dependent increase in C58G transcript levels. However, there is also a decrease in MLP-C58G protein levels due to proteolytic degradation as a consequence of partial protein unfolding [44,53]. Protein destabilization has also been found for L44P through computational in silico studies [57]. The MLP constructs used for overexpression for confocal imaging contained a C-terminal FLAG-tag, and it should be noted that this tag could affect MLP’s functionality and ability of nuclear translocation [29]. However, it seems unlikely that such effects are critical since we successfully detected FLAG-tagged MLP in the nuclear region. Additionally, the FLAG-tagged MLP mouse is able to rescue the DCM phenotype of the MLP KO mouse [58], suggesting MLP is still functional with a C-terminal tag, even if in a monomeric form. The increase in atrial natriuretic peptide (ANF) and brain natriuretic peptide (BNP) accompanying the MLP KO mice is also reduced to baseline when these animals are crossed with the FLAG-tagged MLP [58].

In H9c2 cells, most of the MLP existed in the monomeric form, which increased upon differentiation. MLP has previously been found to translocate to the nucleus in response to stretch, activating ribosomal protein S6 (RPS6) in neonatal rat cardiomyocytes, associated with an increase in protein synthesis and an increase in cell size and hypertrophy development [29]. It is, therefore, likely that the high levels of monomeric MLP present upon differentiation are located in the nucleus, where the protein participates in promoting cell growth and, consequently, the differentiation process.

We also investigated the oligomerization of recombinant MLP-WT and mutated proteins. MLP-WT had a ~25% distribution of each oligomer and monomer. In mutated MLP, however, we observed an increase in monomeric MLP at the expense of trimeric and tetrameric MLP. It is possible that the alterations in oligomerization we observed contribute to the disease pathogenesis by increasing the ratio between monomeric to oligomeric MLP. As has been shown before, MLP oligomerization is altered in rat heart disease models, such as aortic banding and myocardial infarction, where an increase in the monomeric MLP is accompanied by a reduction in oligomeric MLP, especially the tetrameric oligomer [29,59]. The same has been shown in human failing hearts, where mainly monomeric MLP is detected, compared to non-failing hearts, where oligomeric MLP is present [29]. Part of the molecular mechanism of MLP-associated cardiomyopathies likely involves a loss of balance in the ratio of monomeric to oligomeric MLP where extranuclear oligomeric MLP is lost, and monomeric MLP in the nucleus is increased. MLP has been suggested to serve a dual role in the heart, in the nucleus for the maintenance/induction of hypertrophy, and in the cytoplasm as a structural component of the various stretch and stress sensor machineries [15,19,40,60].

The exact domain of MLP self-association has been an area for debate. In vitro, we found that MLP-WT self-association was highest at amino acid residues 50–79, covering the end of the LIM1 domain and the NLS, and 125–154, covering the middle of the LIM2 domain. Others have found the C-terminus to be important for MLP oligomerization [29]. In contrast, the N-terminal LIM domain has been a proposed site of self-association via bimolecular fluorescence complementation assays [45]. Our data support the possibility that both the N- and C-terminal ends of MLP are sites of self-association, as well as the nuclear localization signal. We found that all MLP mutations, with the exception of L44P and C58G, had weaker self-associations at aa 50–79 and 125–154, indicating that mutated MLP is more prone to monomerization, supporting our immunoblotting data of the recombinant mutated MLP proteins.

The oligomeric state may largely influence the syndecan-4–MLP interaction. The syndecan-4 binding domains within MLP largely overlapped with the sites of MLP self-association, suggesting that syndecan-4 binding sites may not be accessible for binding unless MLP is in a monomeric form. We found that monomeric and dimeric MLP were located in nuclear-enriched fractions, where the binding was also present. Our data suggests that most MLP mutations had reduced self-association; however, we also found that syndecan-4 had reduced binding to some MLP mutations. Except for the L44P mutation, it is, therefore, likely that the reduced syndecan-4 binding affinity was caused by the MLP mutation itself and not by alterations in the binding domain availability. However, syndecan-4–MLP binding when MLP is mutated should be tested in vivo to confirm this hypothesis.

Interestingly, syndecan-4 and MLP share multiple binding partners, including α-actinin and the calmodulin-regulated phosphatase, calcineurin [2,61,62], and multiple groups have postulated that MLP aids in anchoring calcineurin to the Z-discs [62,63,64]. Both syndecan-4 and MLP affect the function of PKCα where syndecan-4 binds PKCα [2], and this binding is reduced when syndecan-4 is phosphorylated at serine 179 [2]. In contrast, MLP is involved in the autophosphorylation level of PKCα [65]. The double KO of PKCα and MLP rescue the DCM phenotype observed in MLP KO mice [66], potentially due to MLP being an inhibitor of PKCα. Additionally, MLP is a potential downstream target of PKCα where HCM-associated mutations in MLP exhibit reduced phosphorylation and DCM-association mutations have increased phosphorylation by PKCα [65]. The potential involvement of syndecan-4 in the relationship between PKCα and MLP is an interesting area for further study. Therefore, although we find binding between syndecan-4 and MLP, specifically in nuclear-enriched fractions, we cannot discard the possibility that the two proteins are part of larger protein complexes together elsewhere in the cardiomyocyte.

To conclude, syndecan-4 and MLP interact in nuclear-enriched fractions of adult rat cardiomyocytes. Furthermore, although H9c2 cells express both endogenous syndecan-4 and MLP, these do not interact in baseline conditions, after ISO stimulation, or upon differentiation. Independent of syndecan-4, recombinant MLP-WT had an approximate 25% dispersion between the monomeric and three oligomeric forms, and this ratio was altered when MLP was mutated, yielding a higher level in monomeric MLP at the expense of trimeric and tetrameric MLP. Lastly, the domains of self-association aiding in the oligomerization of MLP likely involve the LIM1, NLS, and LIM2 domains, largely overlapping with the syndecan-4–MLP binding domains. Whether syndecan-4 aids in stabilizing MLP in the nuclear region or, alternatively, preventing MLP from binding to its target transcription factors are compelling possibilities that warrant further studies.

## Data Availability

The data presented in this study are available upon request from the corresponding author.

## Supplementary Materials

The following supporting information can be downloaded at: https://www.mdpi.com/article/10.3390/cells13110947/s1, Figure S1: MLP epitope mapping, co-precipitation of MLP with syndecan-4 in cardiomyocyte-specific syndecan-4 overexpressing mouse LVs, MLP staining pattern, syndecan-4 antibody-blocking experiments and subcellular fraction enrichment analysis; Figure S2: No co-precipitation of endogenous syndecan-4 and MLP in H9c2 cells, lack of structural organization of sarcomeric α-actinin, ponceau staining, subcellular fraction enrichment analysis, syndecan-4 antibody-blocking experiment, validation of differentiation, no co-precipitation of endogenous syndecan-4 and MLP in HL-1 cells, but co-precipitation of endogenous syndecan-4 and MLP in neonatal rat cardiomyocytes; Figure S3: Negative control images of primary rat cardiomyocytes and H9c2 cells; Table S1: Percentage values ± SEM of monomers, dimers, trimers and tetramers of recombinant MLP-WT and MLP mutated proteins shown in Figure 5B.

## Abbreviations

CFL2: cofilin-2; DCM: dilated cardiomyopathy; HCM: hypertrophic cardiomyopathy; ISO: Isoprenaline; LV: left ventricle; MLP: Muscle LIM protein; NLS: nuclear localization signal; RA: all-trans-retinoic acid; SDC4: syndecan-4; SDC4_cyt_: cytoplasmic domain of syndecan-4; WT: wild-type.
