# Supplementary material for: Exploring Syndecan-4 and MLP and Their Interaction in Primary Cardiomyocytes and H9c2 Cells
Source: Cells. 2024 May 30;13(11):947. doi: 10.3390/cells13110947 (PMC11172336; doi:10.3390/cells13110947)
Supplement: Supplementary file 1 [file cells-13-00947-s001.zip › cells-2957253-supplementary material.pdf]

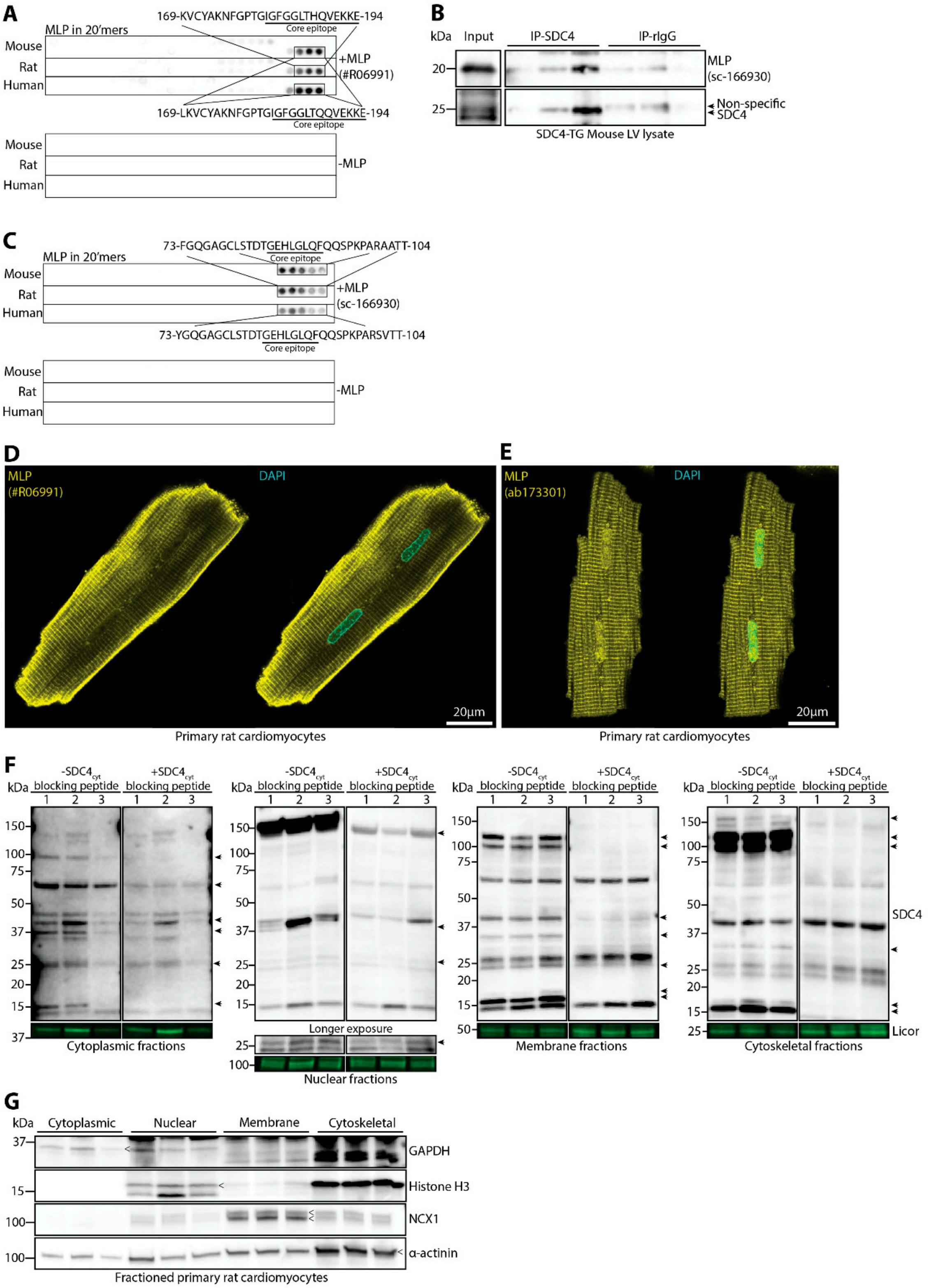

Supplementary Figure S1. MLP epitope mapping, co-precipitation of MLP with syndecan-4 in cardiomyocyte-specific syndecan-4 overexpressing mouse LVs, MLP staining pattern, syndecan-4 antibody-blocking experiments and subcellular fraction enrichment analysis.

(**A**) 20'mer overlapping peptides of mouse, rat and human MLP overlaid with a custom-made antibody against the C-terminal end of MLP (#R06991), used to detect monomeric and oligomeric MLP (upper panel). Boxed regions indicate the binding domain of the antibody to MLP. Amino acids

IGFGGLTQQVEKKE was recognized as the core epitope of human and mouse MLP, and IGFGGLTHQVEKKE in rat MLP (n=2). (**B**) Immunoprecipitation using anti-syndecan-4 (KY/8.2) in adult cardiomyocyte-specific syndecan-4 overexpressing mouse LV lysates. Co-precipitation of MLP was detected with an antibody against MLP (specificity shown in Suppl. Fig. 1C). Non-relevant rat IgG was used as a negative control (n=3 mouse hearts). Syndecan-4 and non-specific precipitation are indicated by arrows on the right. Lysate input is shown on the left. (**C**) 20'mer overlapping peptides of mouse, rat and human MLP overlaid with a commercially available MLP antibody (sc-166930). Boxed regions indicate the binding domain of the antibody to MLP. Amino acids GEHLGLQF was recognized as the core epitope in all species. Identical membranes without the primary antibody were used as a negative control (lower panels in A-B) (n=2). (**D-E**) Representative immunofluorescent images of different MLP antibodies (yellow) (C, #R06991 and D, ab173301) in freshly isolated adult rat primary cardiomyocytes. The cell nuclei were stained with DAPI (cyan). Negative control images are shown in Suppl. Fig 3A. (**F**) The specificity of syndecan-4 positive bands detected in subcellular enriched fractions in Fig. 1C was analyzed using a blocking peptide containing the epitope of the syndecan-4 antibody. Membranes probed with the antibody, pre-incubated with the blocking peptide, are shown in the right panel of each fraction, and membranes probed with the antibody only on the left. Syndecan-4 specific bands are annotated with arrows on the right. A longer exposure image of the 25 kDa bands in the nuclear-enriched fractions is included. Equal loading of duplicate samples was analyzed by licor staining. (**G**) Subcellular compartment markers GAPDH (cytoplasm), histone H3 (nucleus), NCX1 (membrane), and α-actinin (cytoskeleton) were used to assess the enrichment of the fractions used in Fig. 1C (annotated by arrowheads).

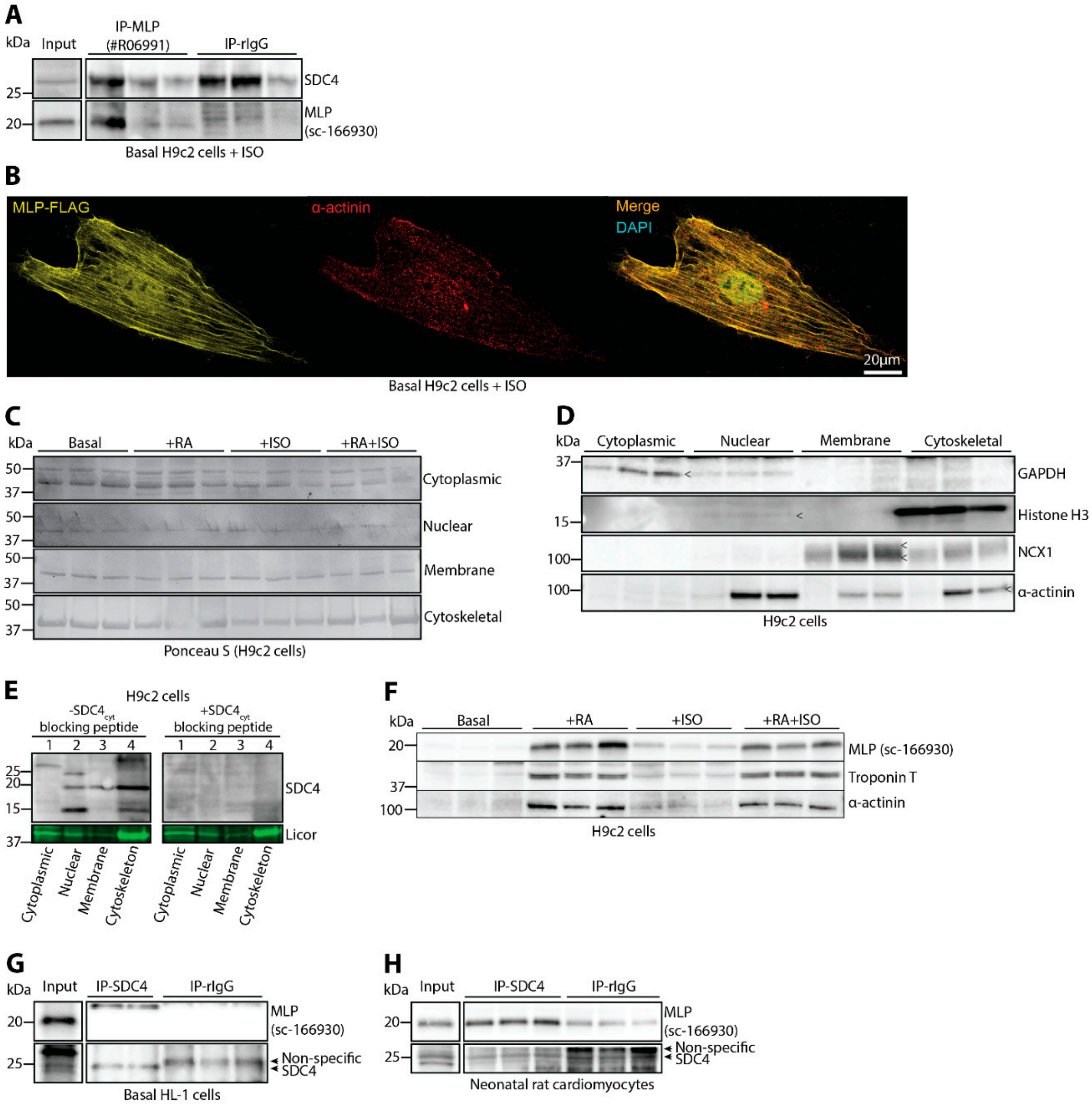

Supplementary Figure S2. No co-precipitation of endogenous syndecan-4 and MLP in H9c2 cells, lack of structural organization of sarcomeric α-actinin, ponceau staining, subcellular fraction enrichment analysis, syndecan-4 antibody-blocking experiment, validation of differentiation, no co-precipitation of endogenous syndecan-4 and MLP in HL-1 cells, but co-precipitation of endogenous syndecan-4 and MLP in neonatal rat cardiomyocytes.

(**A**) Immunoprecipitation of endogenous MLP in isoprenaline (ISO) stimulated H9c2 cells, harvested in lysis buffer. Co-precipitation of SDC4 was assessed with an antibody against the cytoplasmic tail of syndecan-4. Lysate inputs are given in the left panels (n=6). (**B**) Representative immunofluorescence of MLP-FLAG (yellow), α-actinin (red) and DAPI (cyan) in H9c2 cells at baseline conditions treated with 25 µM ISO. Negative control images are shown in Suppl. Fig 3B. (**C**) Ponceau S staining used to verify equal protein loading of immunoblot membranes shown in Fig. 3C and 3E. (**D**) Subcellular compartment markers GAPDH (cytoplasm), histone H3 (nucleus), NCX1 (membrane), and α-actinin (cytoskeleton) were used to assess the enrichment of the fractions used in Fig. 3C and 3E (annotated by arrowheads). Basal, +RA, and +RA+ISO samples from the cytoplasm, nuclear, membrane and cytoskeletal-enriched fractions were analyzed. (**E**) The specificity of syndecan-4 bands detected in the

subcellular enriched fractions of H9c2 cells in Fig. 3E (left panel) were confirmed using a blocking peptide (DLGKKPIYKKAPTN) containing the epitope of the syndecan-4 antibody (#429716) (right panel). Samples from the cytoplasm (+RA+ISO), nuclear (+ISO), membrane (+RA), cytoskeleton (basal) were analyzed. Equal loading of duplicate samples was shown by licor staining. (**F**) Immunoblot analysis of MLP, troponin T and sarcomeric α-actinin in H9c2 cells +/-RA +/- ISO, harvested in RIPA buffer (n=3). (**G-H**) Immunoprecipitation using anti-syndecan-4 (KY/8.2) in (G) HL-1 lysates and (H) neonatal rat cardiomyocyte lysates. Co-precipitation of MLP was detected with an antibody against MLP (specificity shown in Suppl. Fig. 1C). Non-relevant rat IgG was used as a negative control. Syndecan-4 and non-specific precipitation are indicated by arrows on the right. Lysate input is shown on the left.

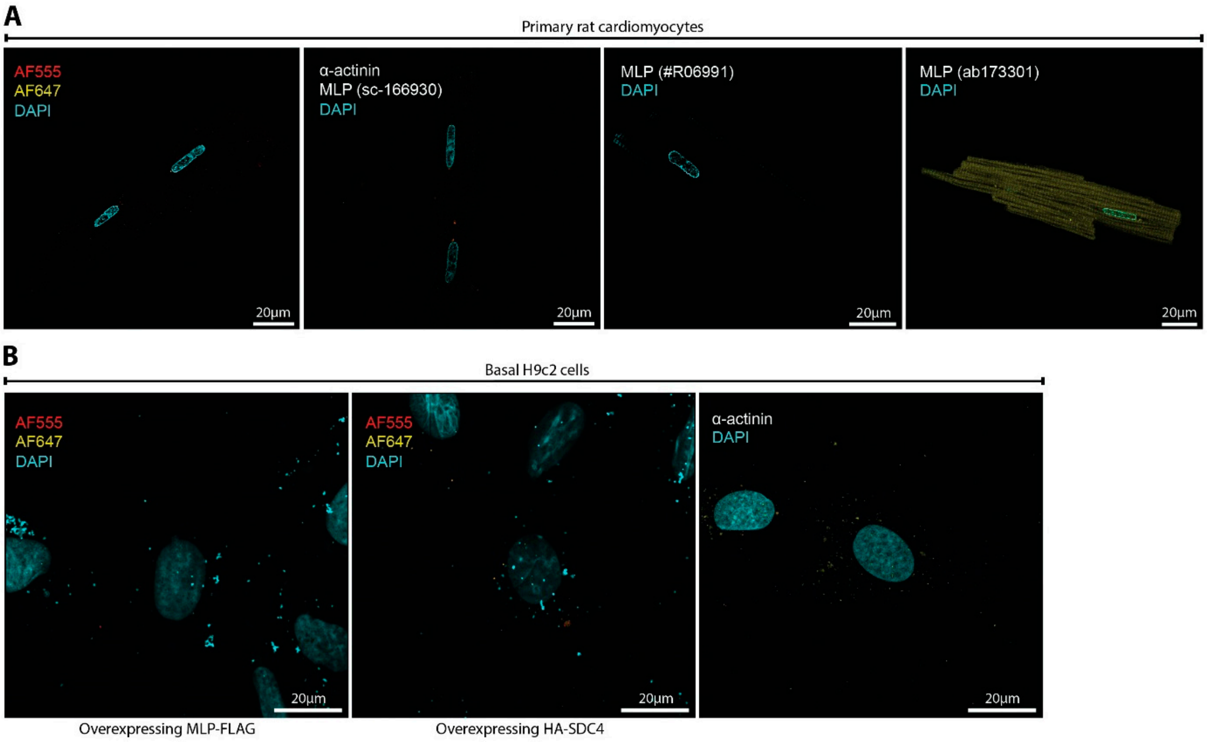

Supplementary Figure S3. Negative control images of primary rat cardiomyocytes and H9c2 cells. (**A**) Representative negative control immunofluorescence of freshly isolated primary rat cardiomyocytes stained with indicated primary or secondary antibodies only, and DAPI. All images were excited with the 555 and 647 nm laser lines. Note the weak autofluorescence of the MLP antibody ab173301, excited at 647 nm. (**B**) Representative negative control immunofluorescence of H9c2 cells stained with indicated primary or secondary antibodies only, and DAPI. All images were excited with the 555 and 647 nm laser lines.

Supplementary Table S1. Percentage values ± SEM of monomers, dimers, trimers and tetramers of recombinant MLP-WT and MLP mutated proteins shown in Fig. 5B. Differences between WT and mutated MLP monomers and each oligomer were tested with Mann-Whitney U tests due to non-normal distribution analyzed by Shapiro-Wilk testing (*p < 0.05, **p < 0.01, ***p < 0.001, ****p < 0.0001).

	WT (n=18)	W4R (n=6)	L44P (n=6)	C58G (n=6)	R64C (n=6)	Y66C (n=6)	K69R (n=6)	G72R (n=6)	Q91L (n=6)
**Tetramer**	17 ± 2	2 ± 1 ****	2 ± 1 ****	3 ± 2 ****	5 ± 2 ****	2 ± 0 ****	6 ± 2 ****	2 ± 1 ****	1 ± 1 ****
**Trimer**	28 ± 6	9 ± 2 ****	9 ± 6 ****	18 ± 7 **	11 ± 3 ****	7 ± 2 ****	11 ± 2 ****	6 ± 1 ****	2 ± 1 ****
**Dimer**	30 ± 8	30 ± 1	36 ± 15	26 ± 3	34 ± 12	24 ± 4 *	25 ± 2 *	22 ± 2 **	14 ± 1 ****
**Monomer**	25 ± 6	59 ± 12 ****	53 ± 15 ***	53 ± 6 ****	50 ± 5 ****	68 ± 6 ****	58 ± 4 ****	70 ± 8 ****	83 ± 5 ****
