# Supplementary material for: Exploring Syndecan-4 and MLP and Their Interaction in Primary Cardiomyocytes and H9c2 Cells
Source: Cells. 2024 May 30;13(11):947. doi: 10.3390/cells13110947 (PMC11172336; doi:10.3390/cells13110947)

Full length blots for figure 1A

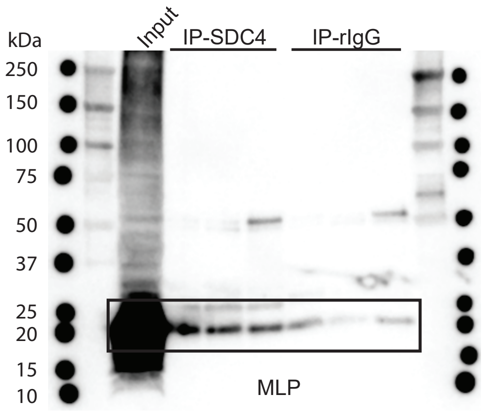

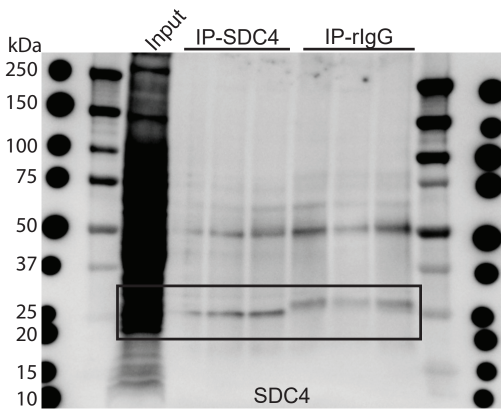

Full length blots for figure 1C

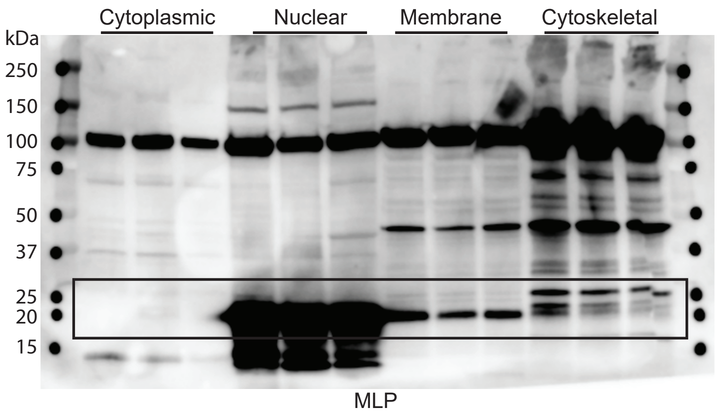

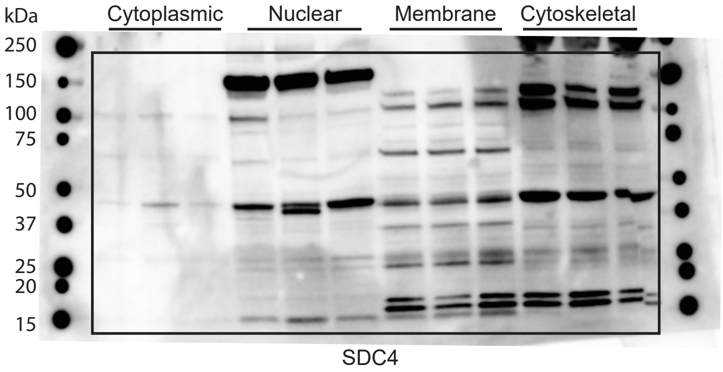

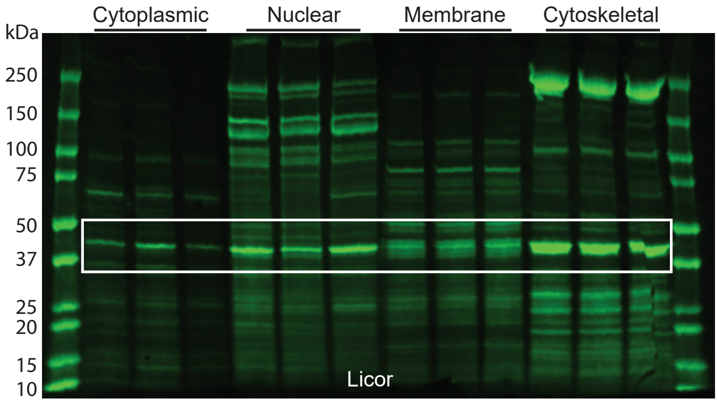

Full length blots for figure 1D

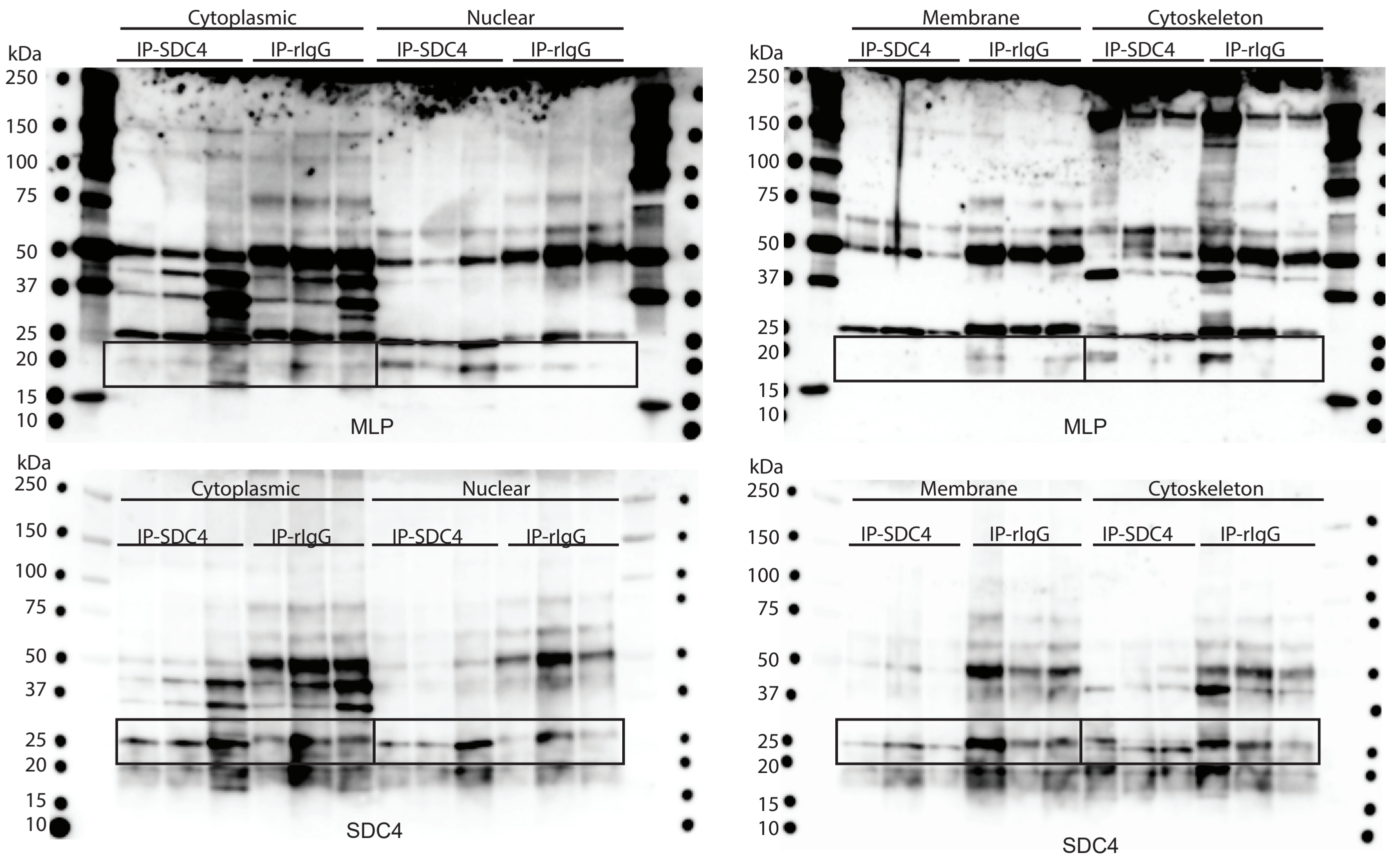

Full length blots for figure 1E

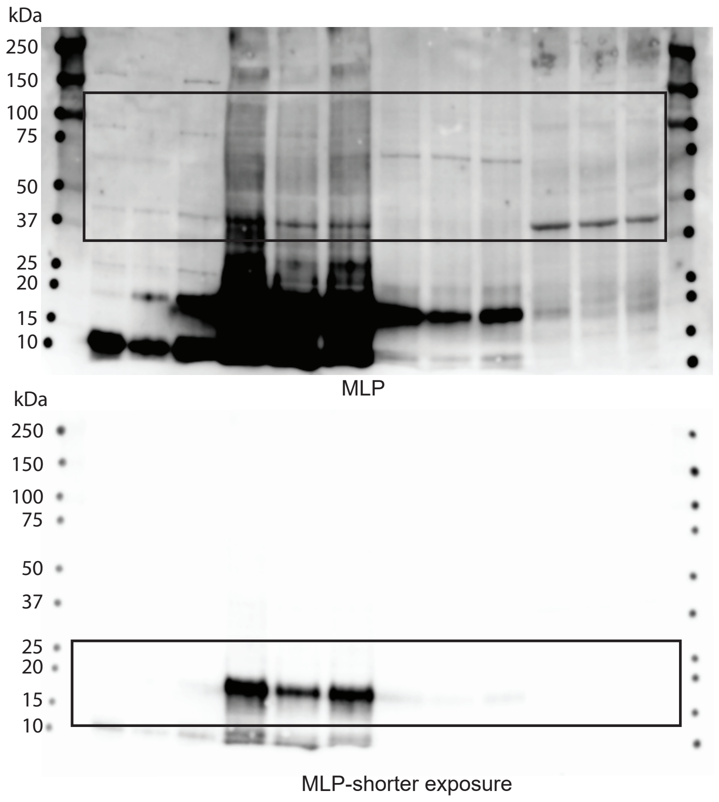

Full length blots for figure 3B

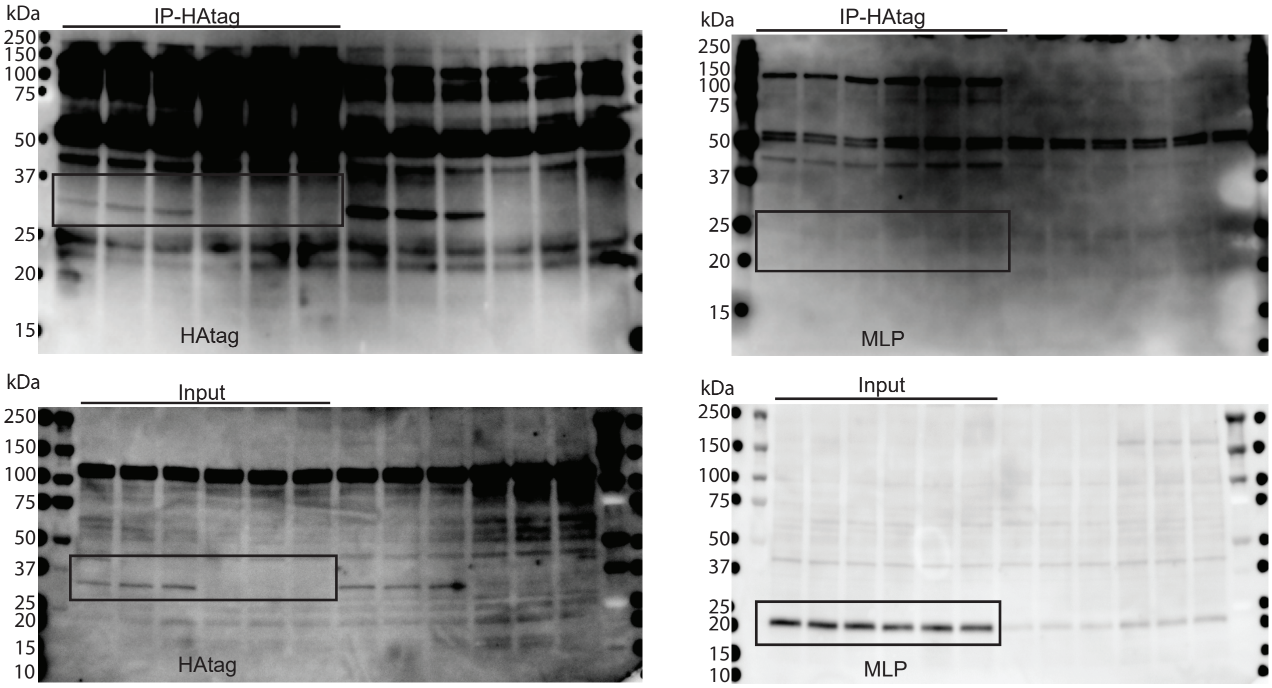

Full length blots for figure 3C

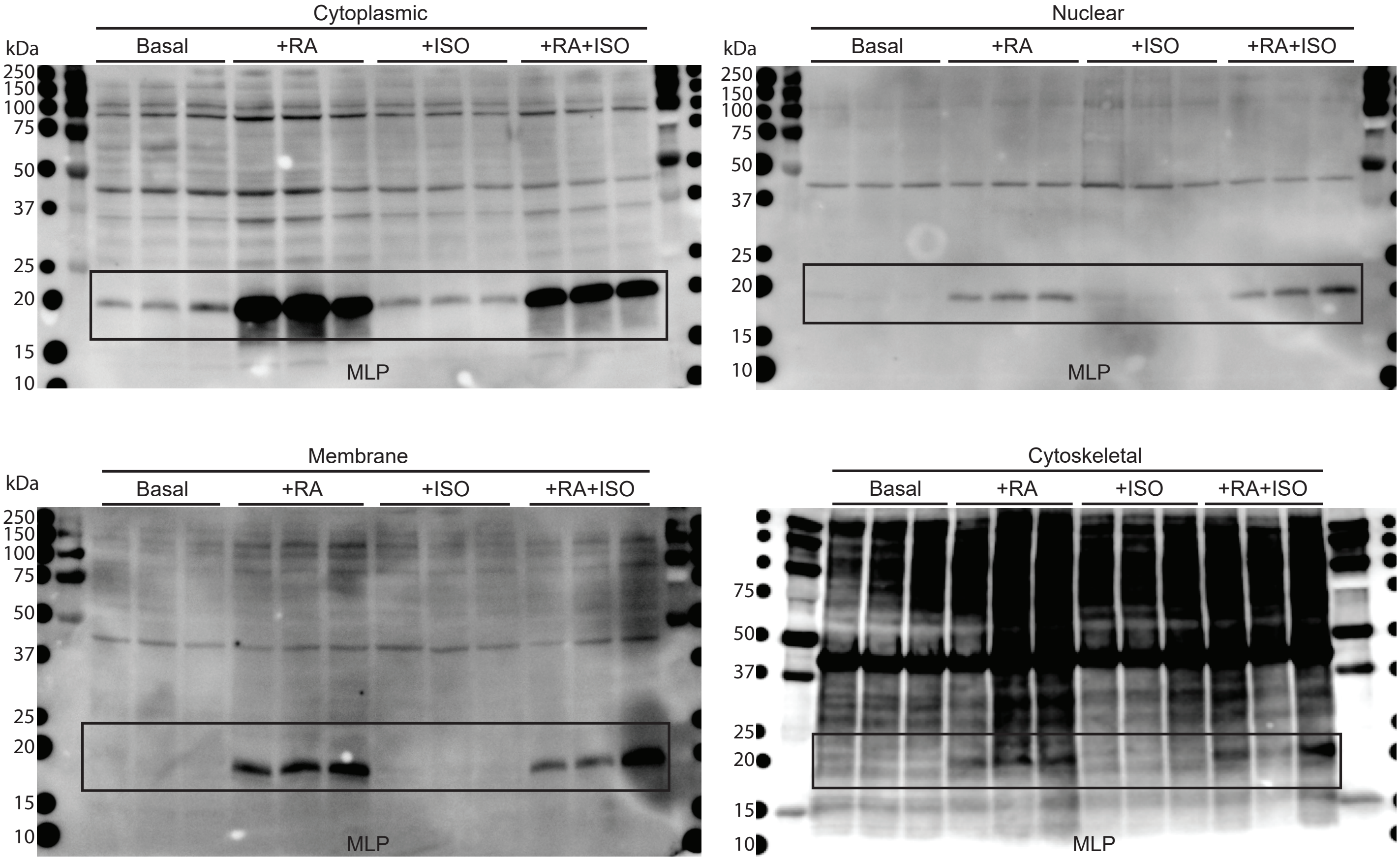

Full length blots for figure 3E

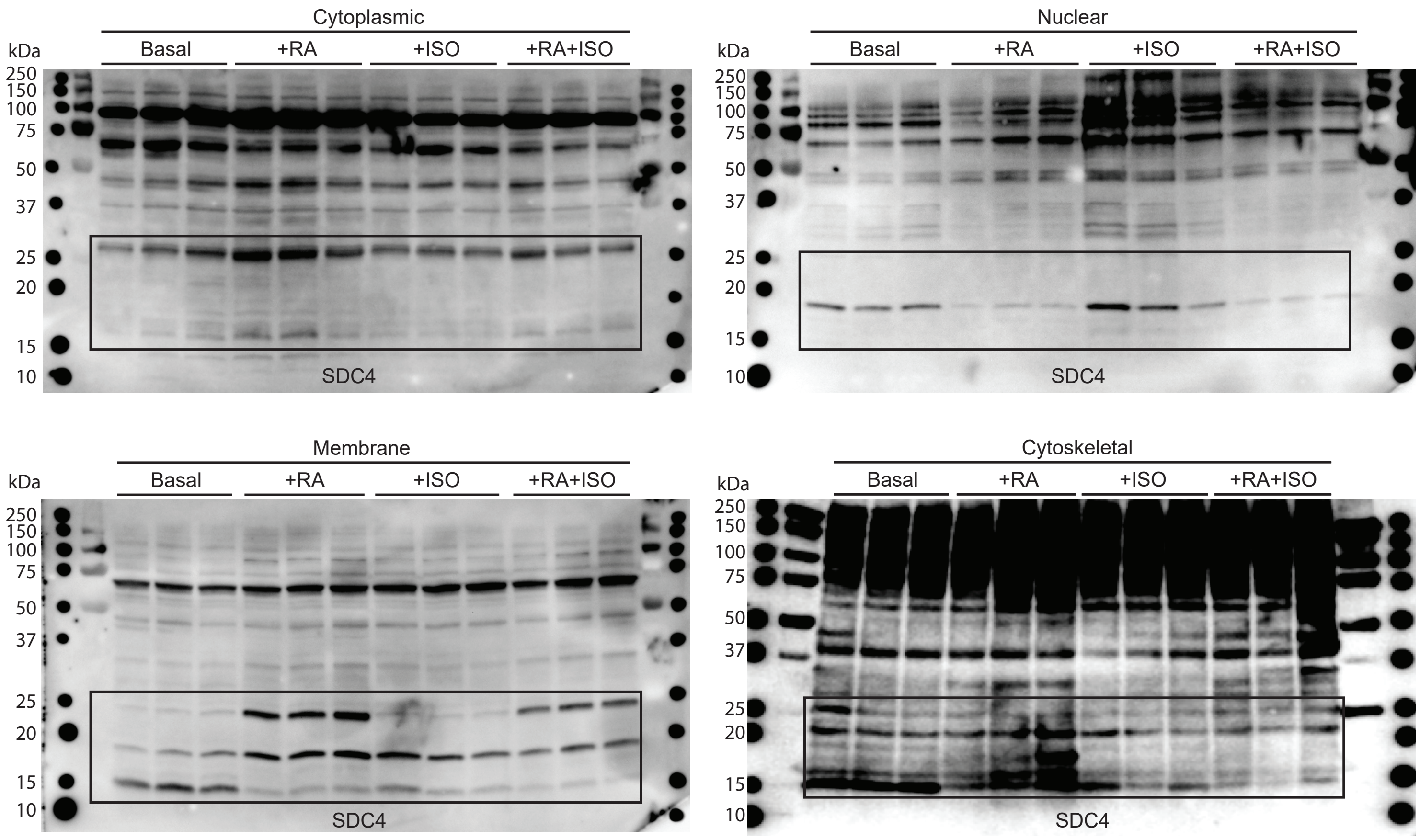

Full length blots for figure 3F

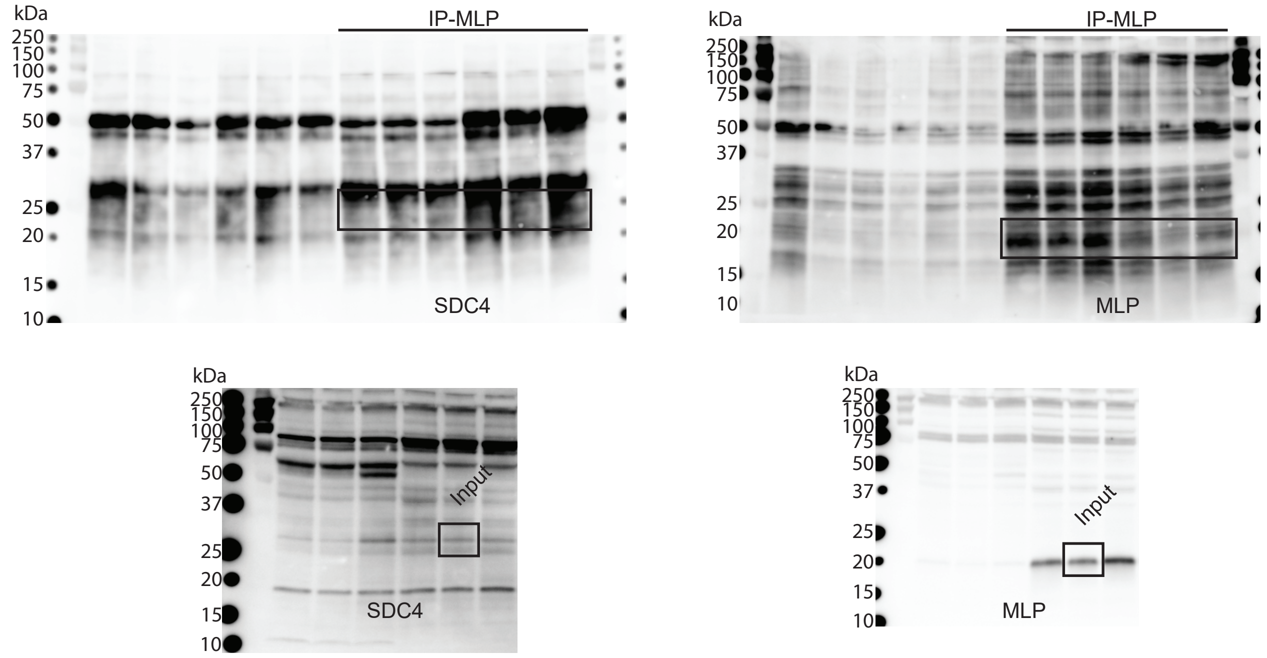

Full length blots for figure 3G

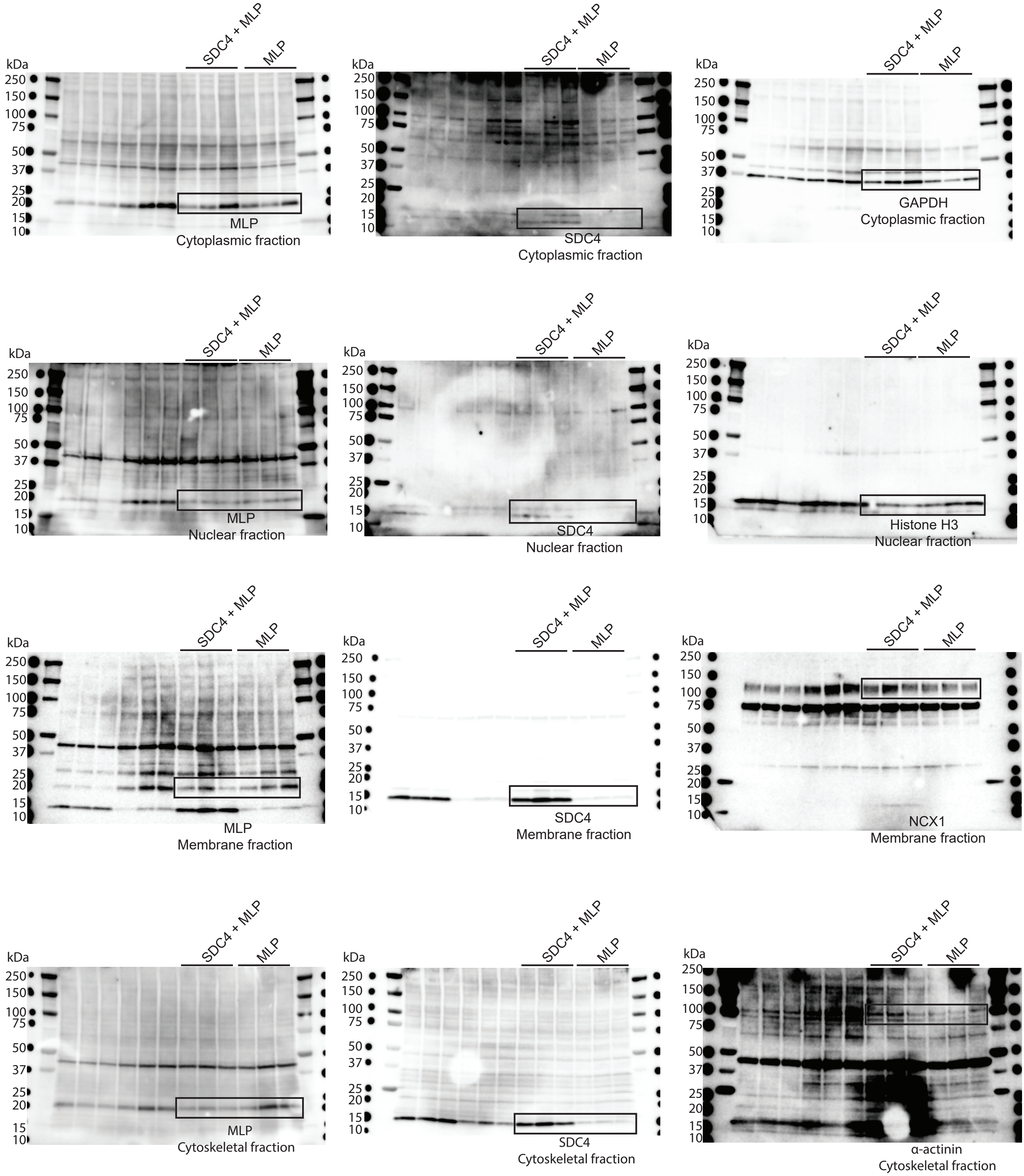

Full length blots for figure 4A.1

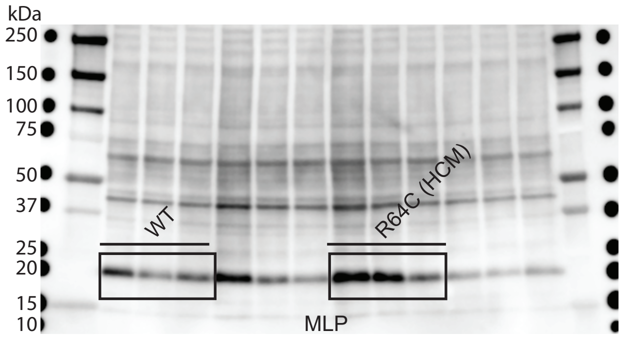

MLP
Cytoplasmic fraction

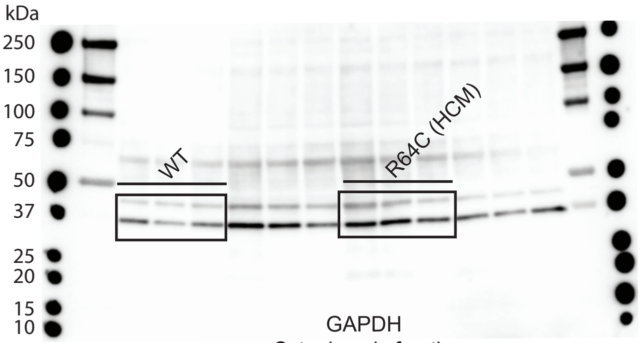

GAPDH
Cytoplasmic fraction

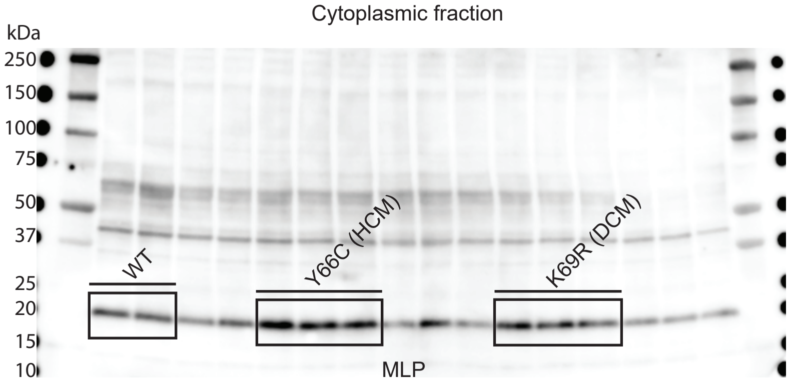

MLP
Cytoplasmic fraction

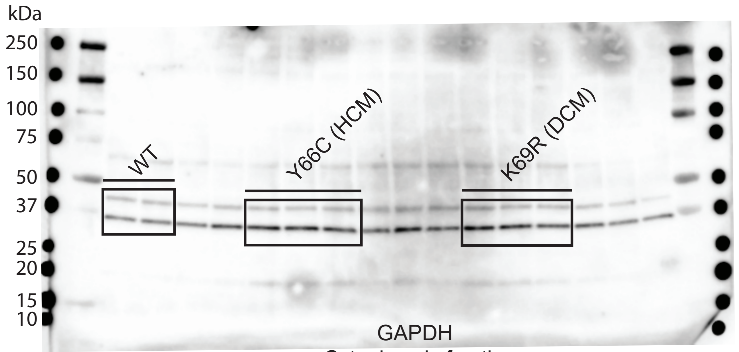

GAPDH
Cytoplasmic fraction

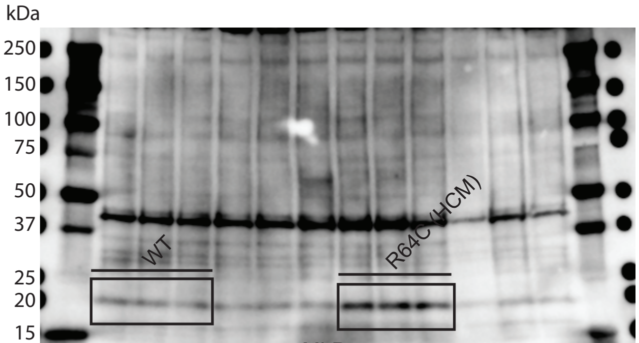

MLP
Nuclear fraction

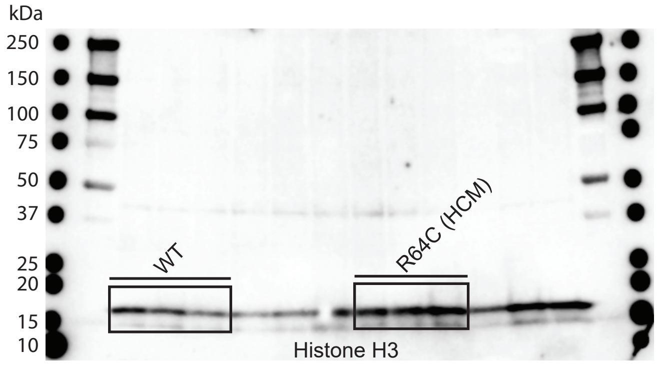

Histone H3
Nuclear fraction

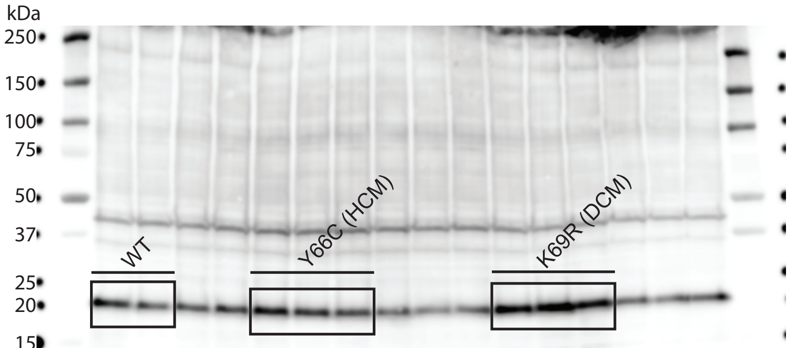

MLP
Nuclear fraction

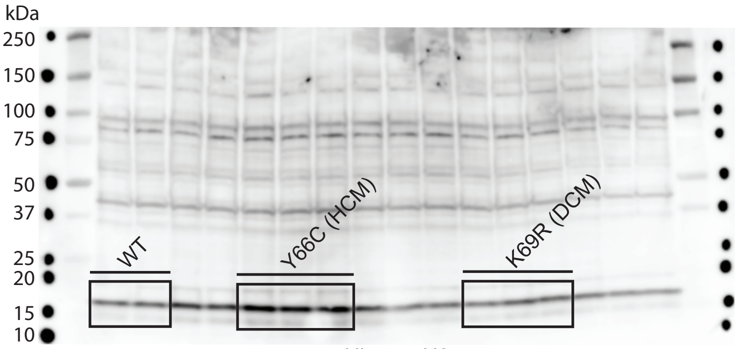

Histone H3
Nuclear fraction

Full length blots for figure 4A.2

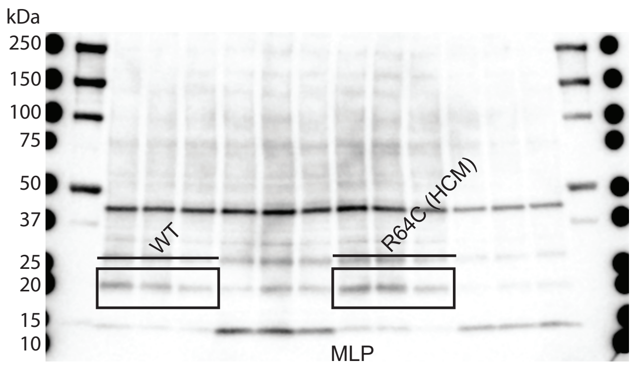

MLP
Membrane fraction

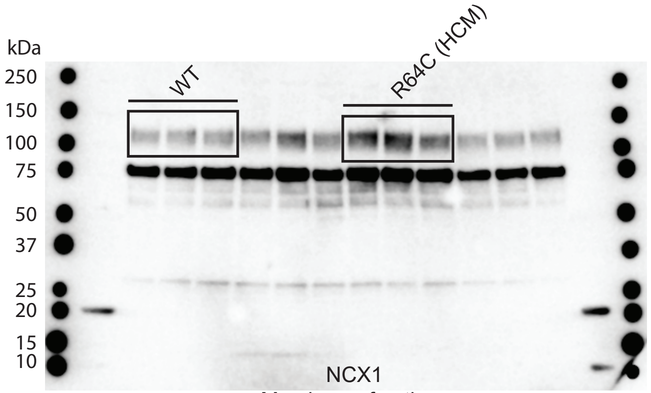

NCX1
Membrane fraction

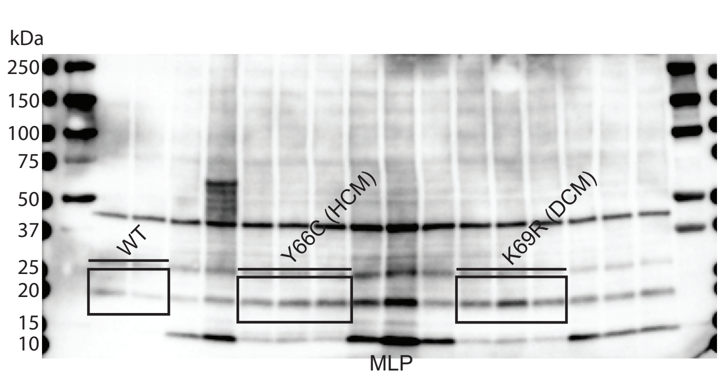

MLP
Membrane fraction

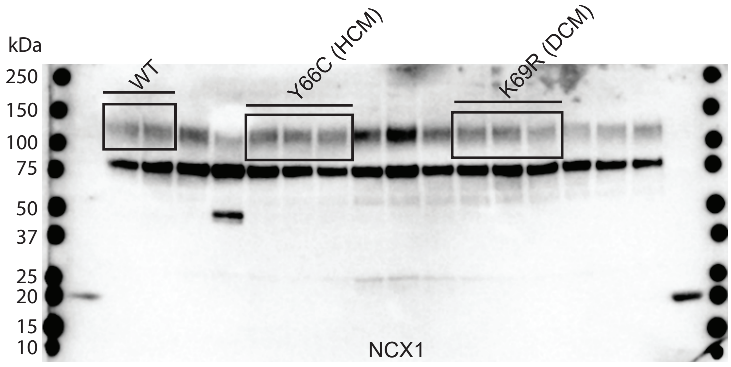

NCX1
Membrane fraction

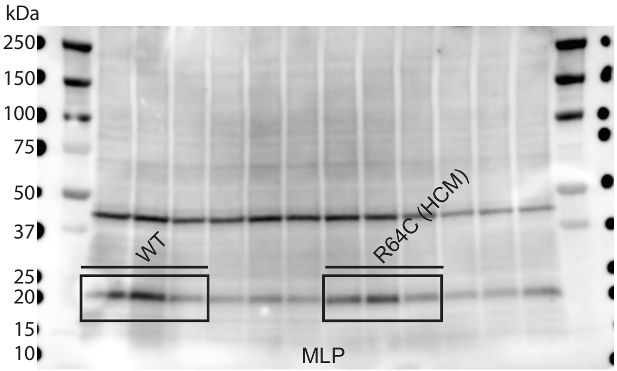

MLP
Cytoskeletal fraction

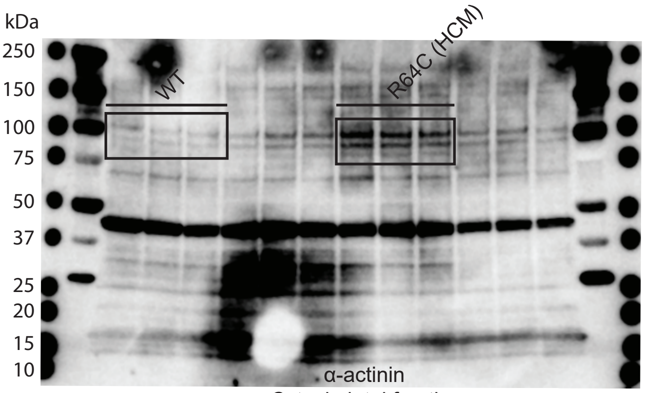

α-actinin
Cytoskeletal fraction

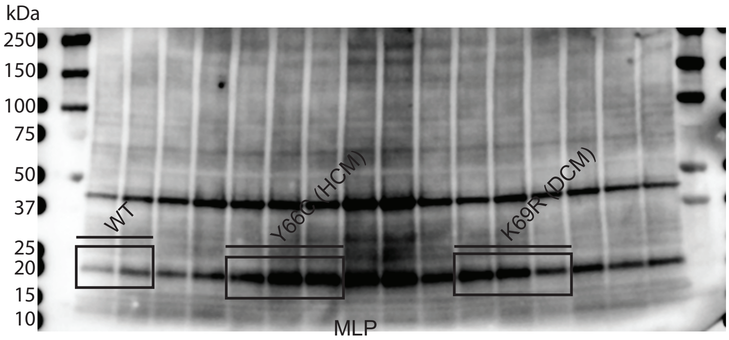

MLP
Cytoskeletal fraction

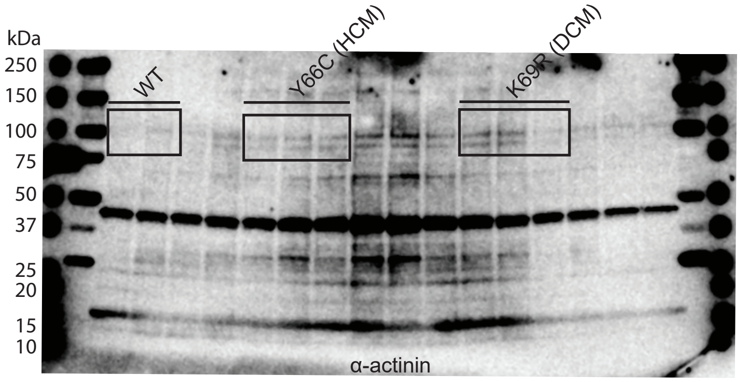

α-actinin
Cytoskeletal fraction

Full length blots for figure 4A.3

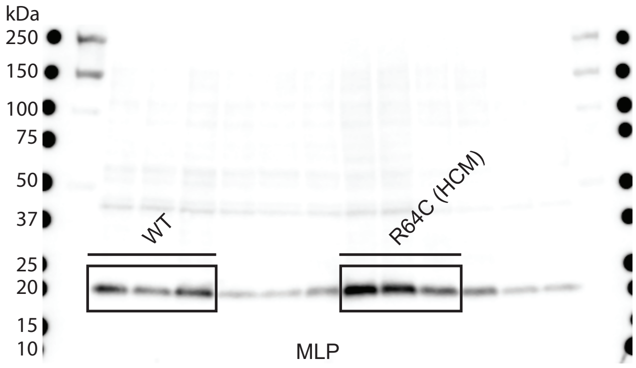

MLP
Total lysates

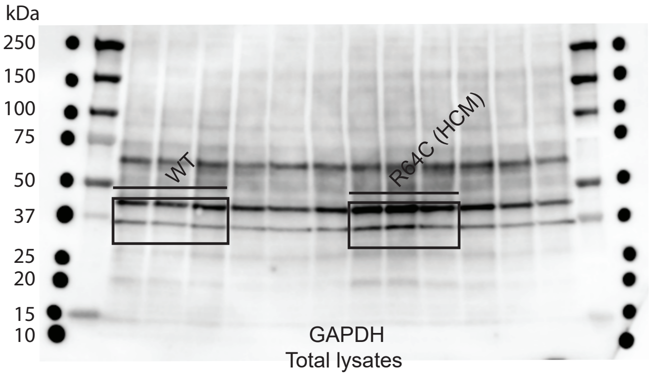

GAPDH
Total lysates

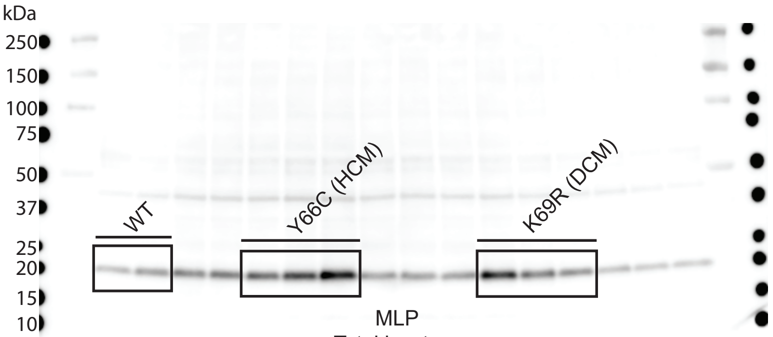

MLP
Total lysates

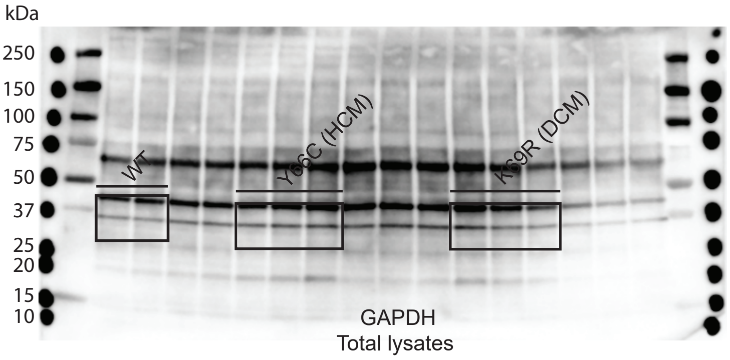

GAPDH
Total lysates

Full length blots for figure 5A

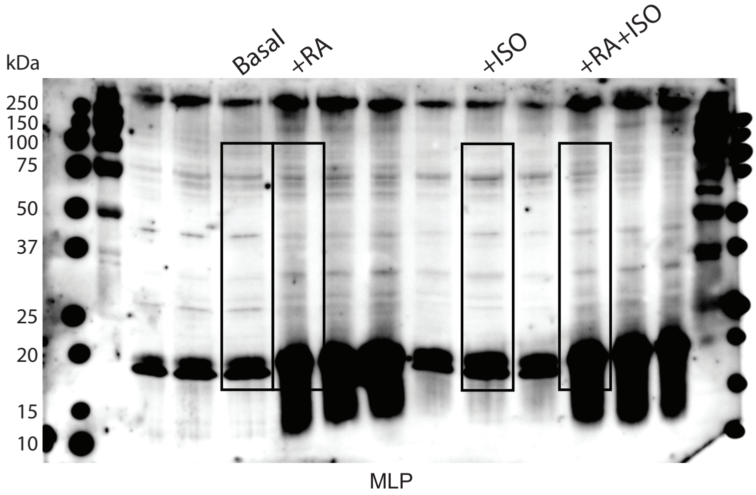

Full length blots for figure 5B

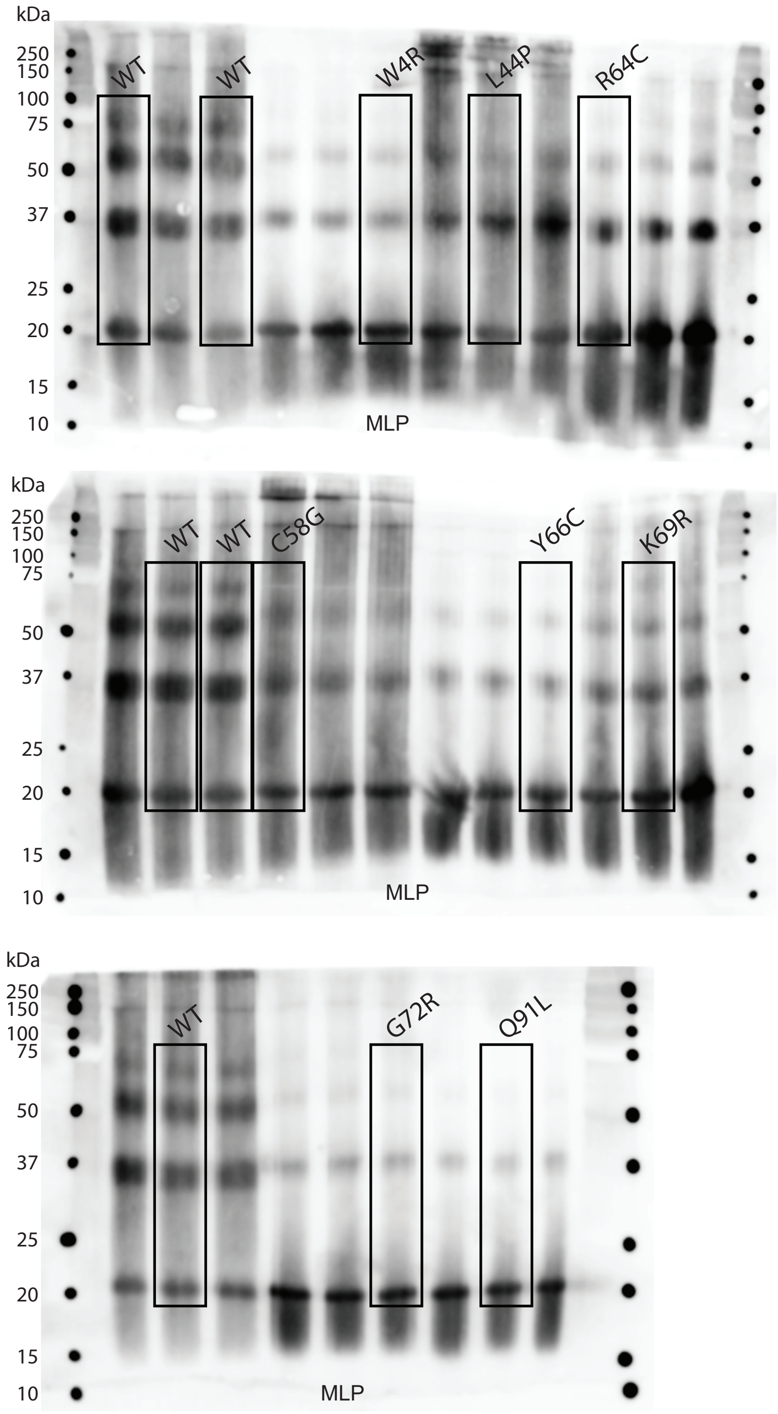

Full length blots for supplementary figure 1B

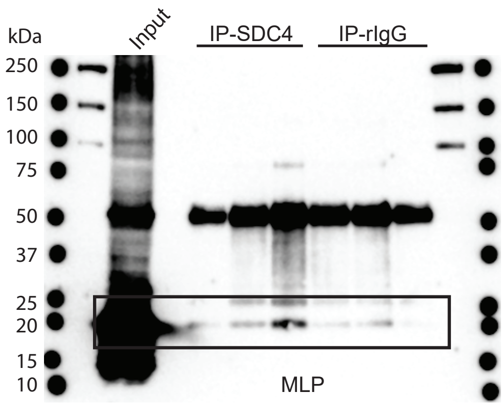

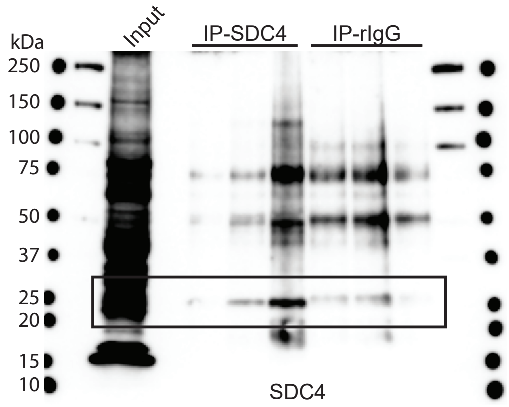

Full length blots for supplementary figure 1F

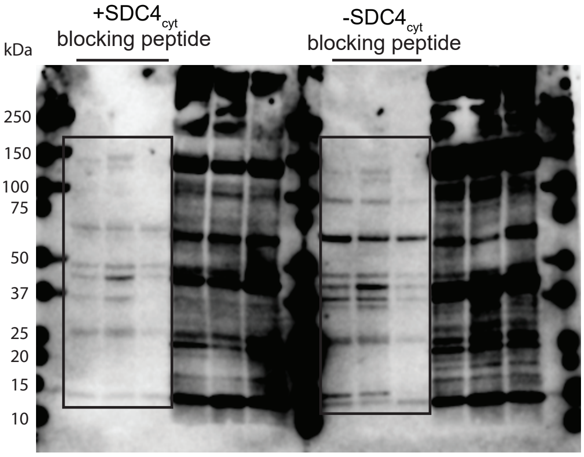

SDC4
Cytoplasmic fraction

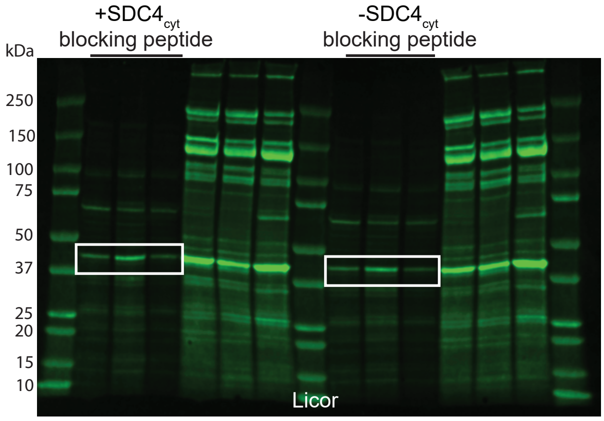

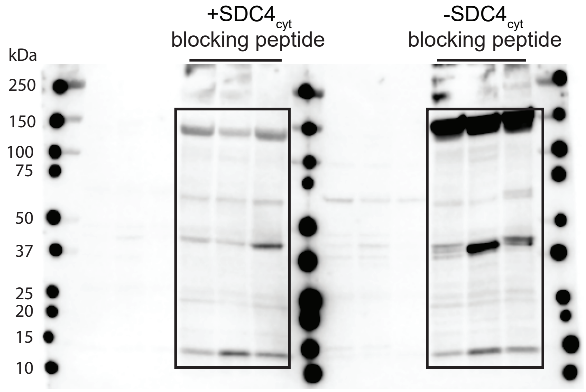

SDC4
Nuclear fraction

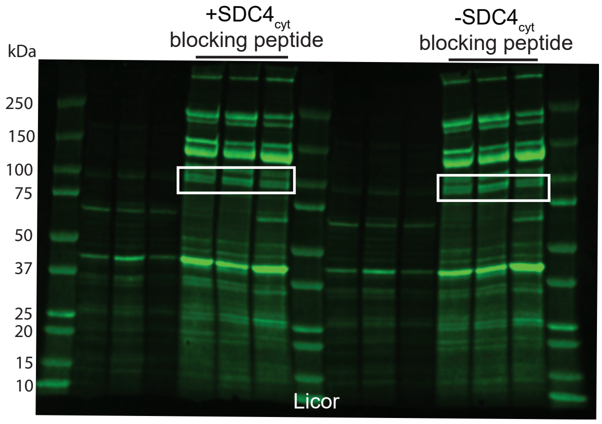

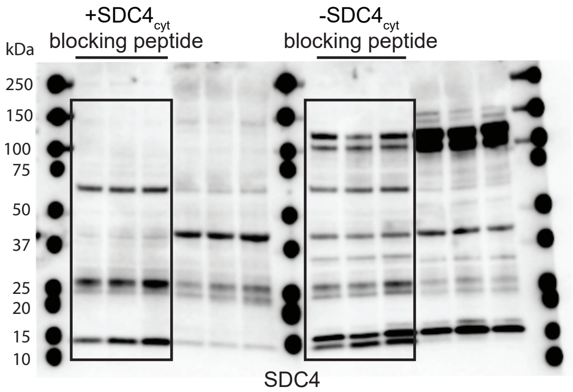

SDC4
Membrane fraction

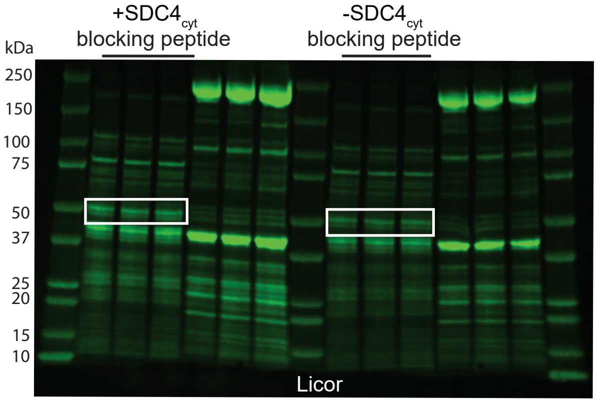

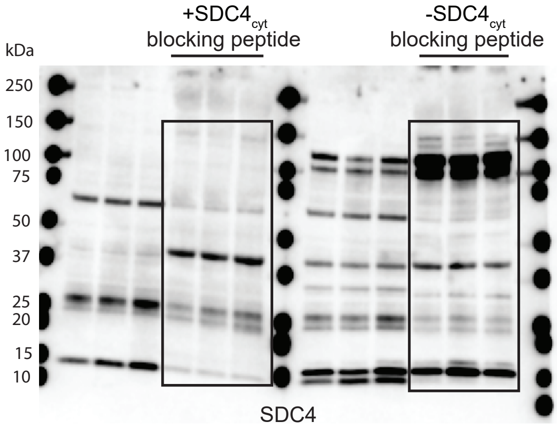

SDC4
Cytoskeleton fraction

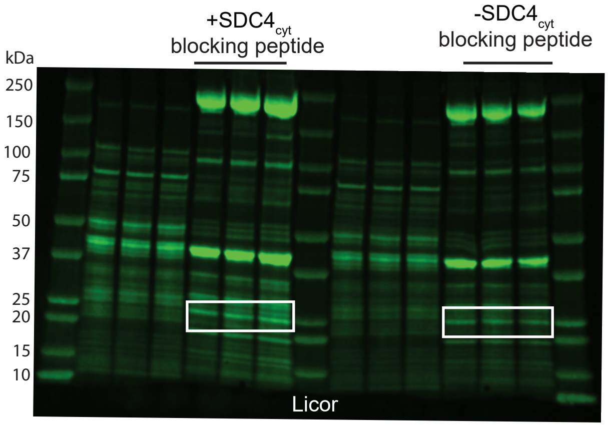

Full length blots for supplementary figure 1G

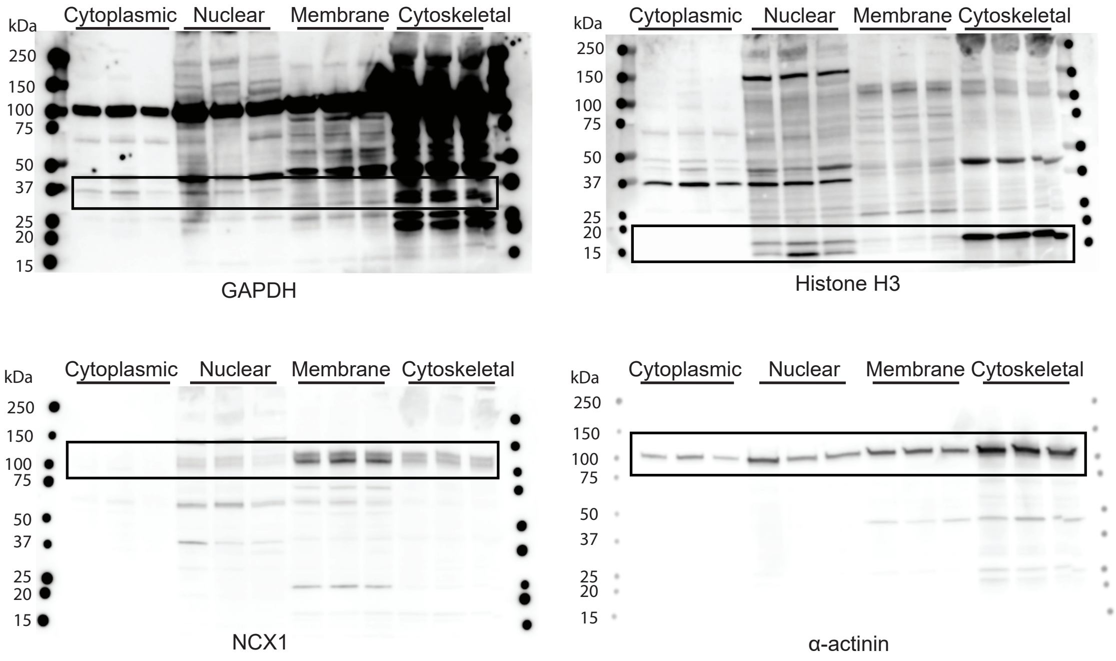

Full length blots for supplementary figure 2A

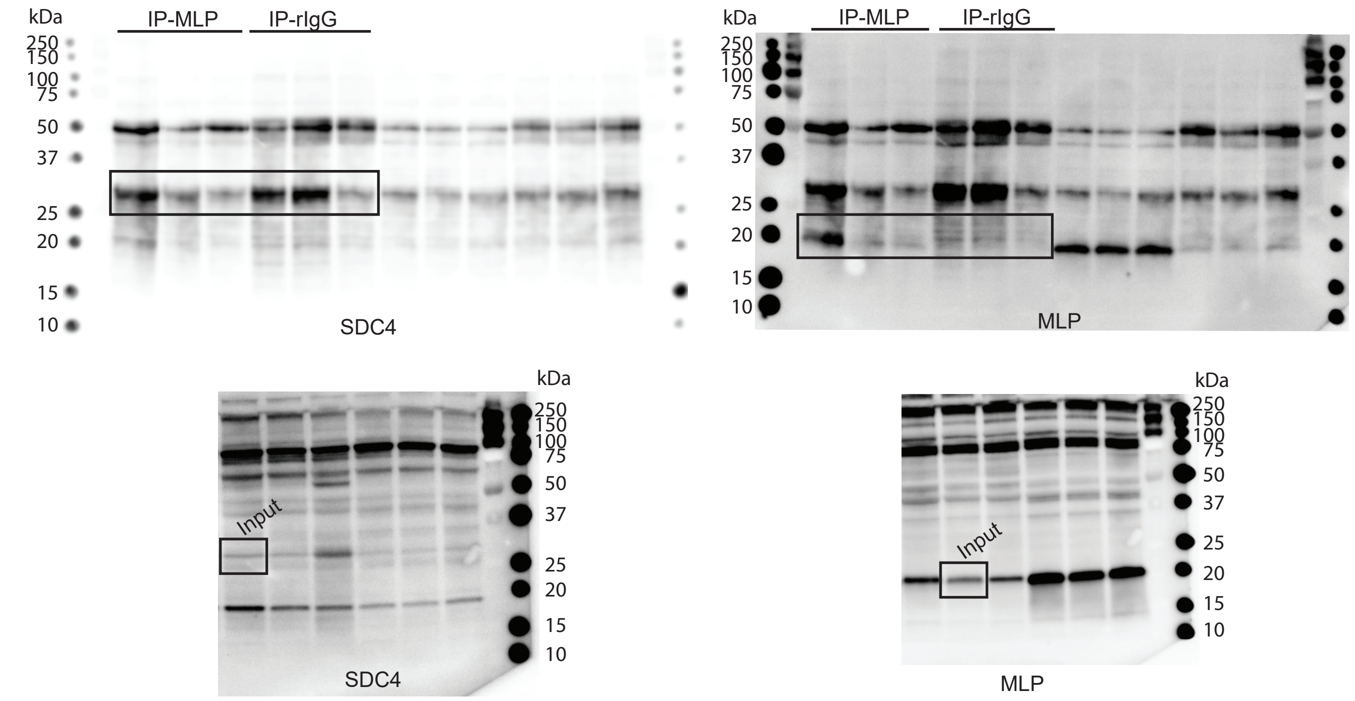

Full length blots for supplementary figure 2C

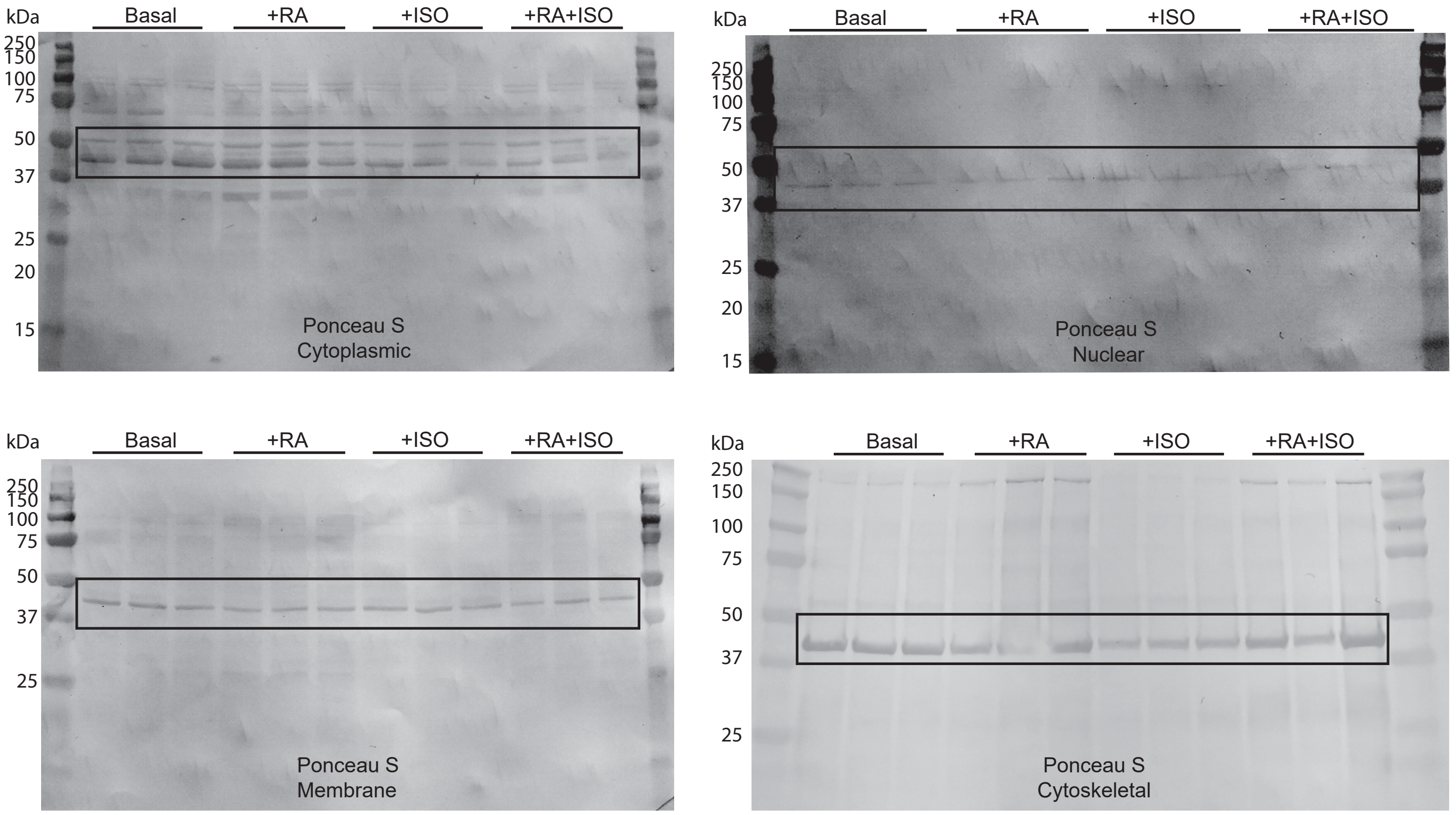

Full length blots for supplementary figure 2D

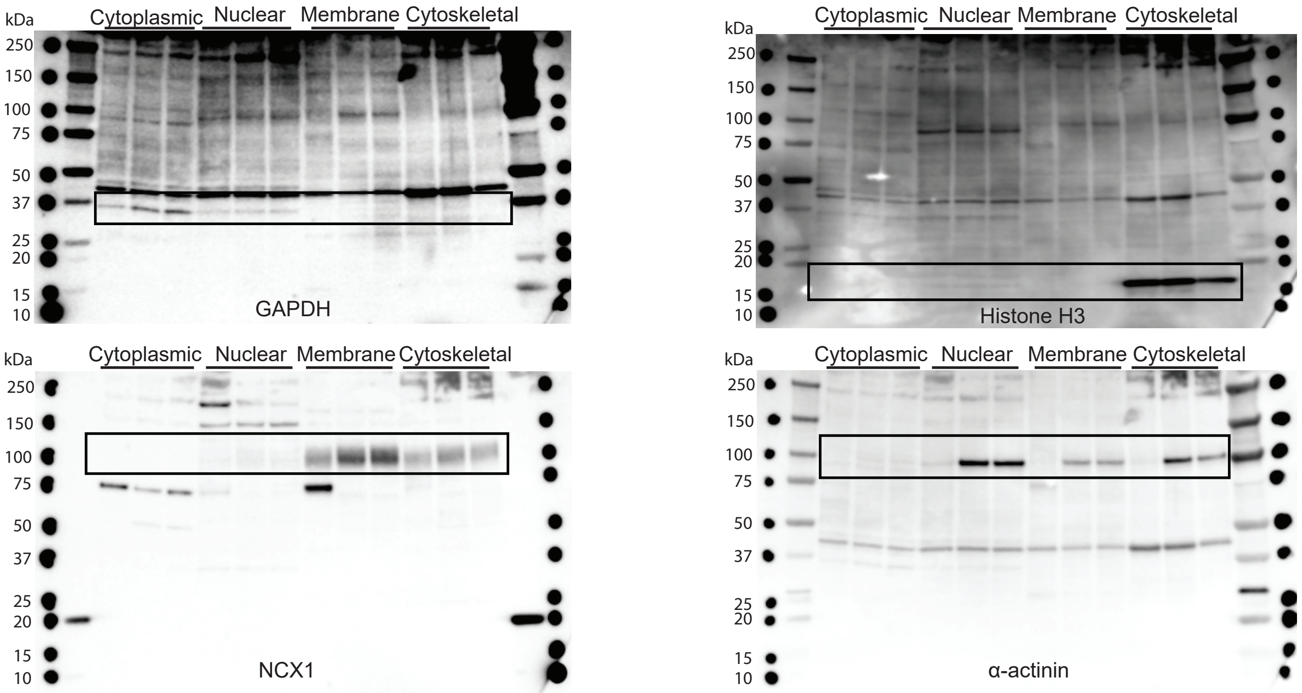

Full length blots for supplementary figure 2E

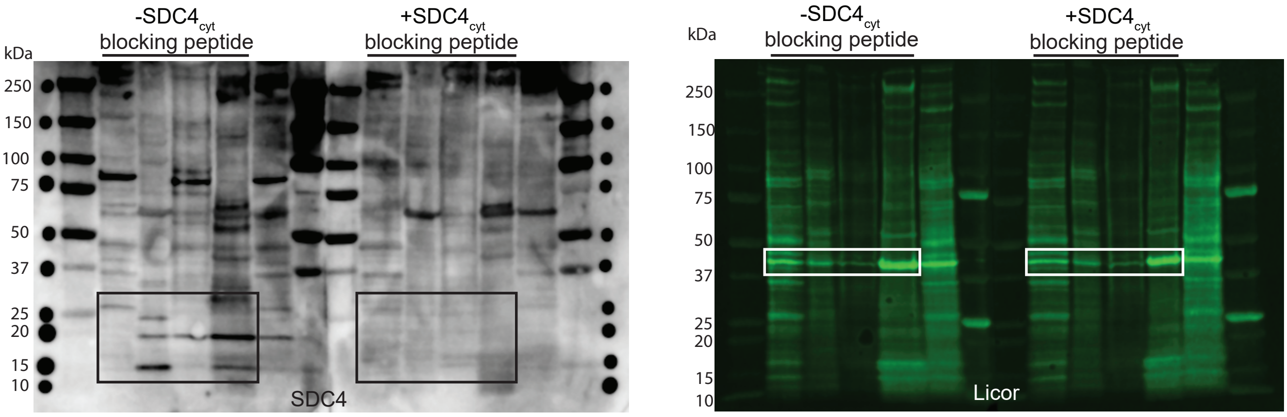

Full length blots for supplementary figure 2F

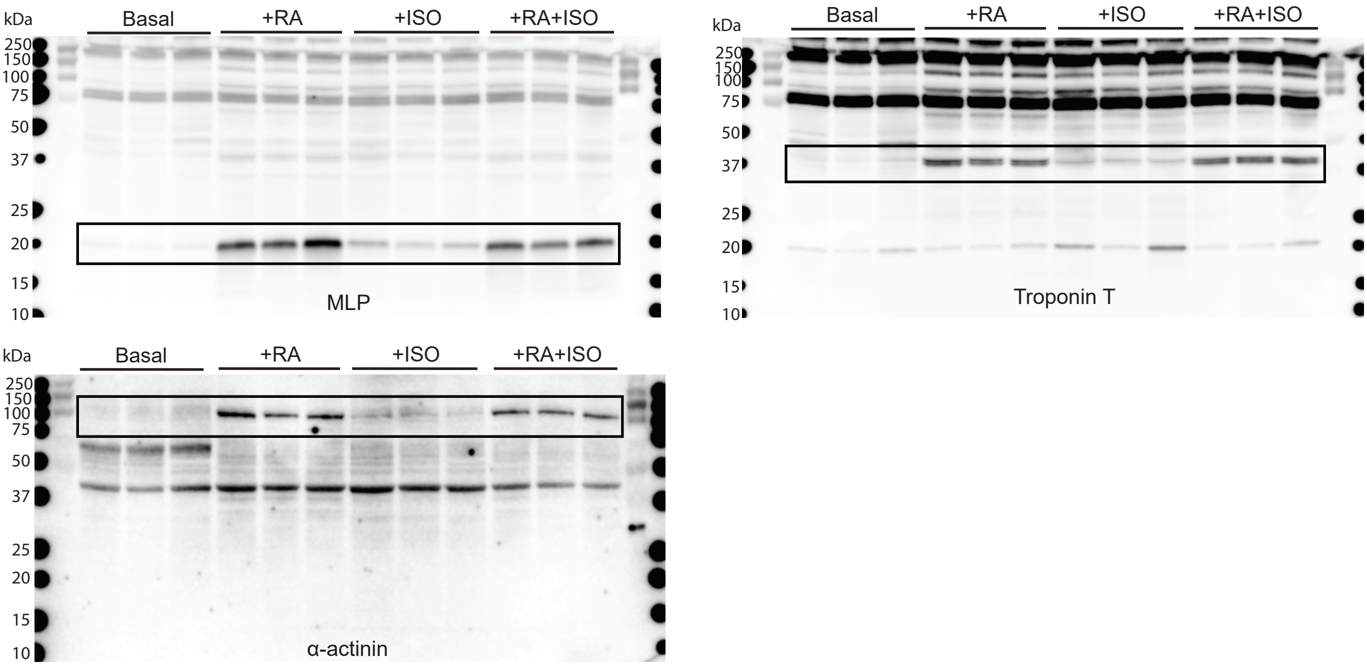

Full length blots for supplementary figure 2G

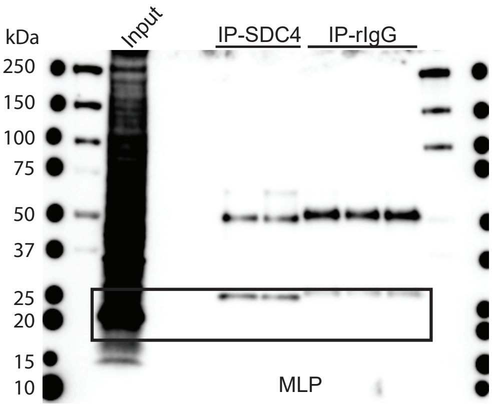

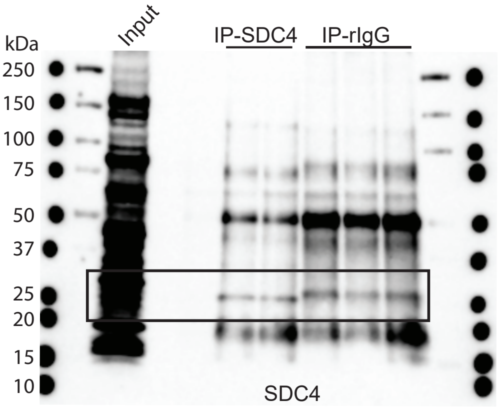

Full length blots for supplementary figure 2H

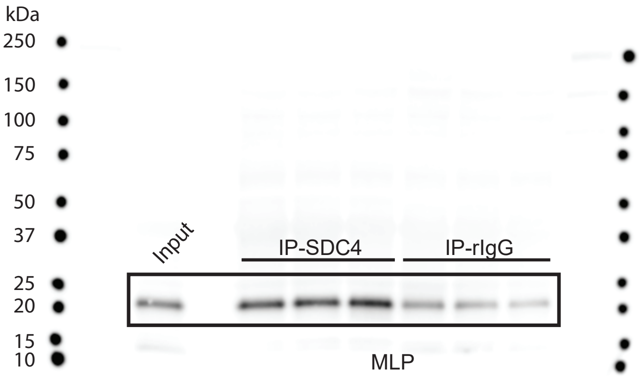

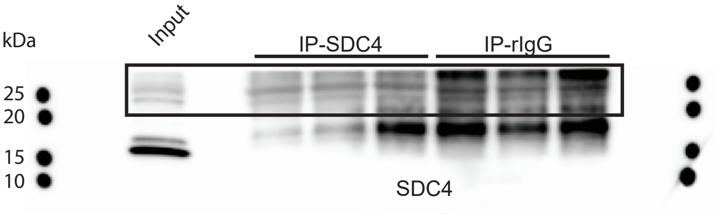

Supplement: Supplementary file 1 [file cells-13-00947-s001.zip › Full-length blots.pdf]
